# Violencia de pareja y búsqueda de ayuda en Chiapas, México: implicaciones para servicios de salud rural comunitaria

**DOI:** 10.18294/sc.2025.5623

**Published:** 2025-11-04

**Authors:** Mercedes Aguerrebere, Ana Cecilia Ortega, Rocío López, Sonia M. Frías

**Affiliations:** 1 Autora de correspondencia. Médica Especialista en Psiquiatría. Consultora en Psiquiatría, Compañeros en Salud México A.C., Partners in Health Mexico. Chiapas, México. Investigadora, Department of Global Health and Social Medicine, Harvard Medical School. Boston, Estados Unidos de América. maguerrebere@pih.org Partners in Health Mexico Compañeros en Salud México A.C. Partners in Health Mexico Chiapas Mexico; 2 Maestra en Desarrollo Internacional. Coordinadora, Programa de Salud Mental, Compañeros en Salud México A.C, Partners in Health Mexico, Chiapas, México. aortega@pih.org Partners in Health Mexico Programa de Salud Mental Compañeros en Salud México A.C Partners in Health Mexico Chiapas Mexico aortega@pih.org; 3 Médica Cirujana. Escuela de Medicina y Ciencias de la Salud. TecSalud, Monterrey, México. rociolopzb@gmail.com TecSalud Escuela de Medicina y Ciencias de la Salud TecSalud Monterrey Mexico rociolopzb@gmail.com; 4 Doctora en Sociología, Centro Regional de Investigaciones Multidisciplinarias, Universidad Nacional Autónoma de México. Morelos, México. sfrias@crim.unam.mx Universidad Nacional Autónoma de México Centro Regional de Investigaciones Multidisciplinarias Universidad Nacional Autónoma de México Morelos Mexico sfrias@crim.unam.mx

**Keywords:** Salud Rural, Violencia de Pareja, Perspectiva de Género, Pobreza, México, Rural Health, Intimate Partner Violence, Gender Perspective, Poverty, Mexico

## Abstract

Desde la perspectiva de género, este estudio examina cómo las mujeres buscan ayuda ante experiencias de violencia de pareja, diferenciando entre dinámicas de terrorismo íntimo y violencia situacional, en una comunidad rural en Chiapas, México. Se realizó un estudio probabilístico entre julio y agosto 2017 en la que participaron 141 mujeres de 15 años o más, utilizando una adaptación de la Encuesta Nacional sobre la Dinámica de las Relaciones en los Hogares (ENDIREH). Además, entre julio y diciembre 2017 se realizaron entrevistas en profundidad a mujeres y líderes locales seleccionados intencionalmente y observación participante. El 59% de las sobrevivientes revelaron la violencia, y solo 7,1% buscó ayuda formal, principalmente quienes vivían terrorismo íntimo. La mayoría buscó ayuda en su familia y 78,9% se sintió apoyada por ella; el apoyo incluyó apoyo emocional, enfrentarse a los agresores y salvaguardar a las mujeres. Emociones como el miedo y la vergüenza, la impunidad, las normas de género tradicionales, los factores socioeconómicos y las redes sociales restringidas inhiben la búsqueda de ayuda. En este contexto, programas de salud comunitaria pueden fortalecer redes sociales y brindar atención.

## Abstract (en)

From a gender perspective, this study examines how women seek help in response to experiences of intimate partner violence, distinguishing between dynamics of intimate terrorism and situational violence in a rural community in Chiapas, Mexico. A probabilistic study was conducted between July and August 2017 with the participation of 141 women aged 15 years and older, using an adaptation of the National Survey on the Dynamics of Household Relationships (ENDIREH). In addition, from July to December 2017, in-depth interviews were conducted with intentionally selected women and local leaders, along with participant observation. Among survivors, 59% disclosed the violence, and only 7.1% sought formal help, mainly those who experienced intimate terrorism. Most sought help from their families, and 78.9% felt supported by them; this support included emotional assistance, confronting aggressors, and protecting women. Emotions such as fear and shame, impunity, traditional gender norms, socioeconomic factors, and restricted social networks inhibit help-seeking. In this context, community health programs can play a key role in strengthening social networks and providing care.

## Introducción

La violencia contra las mujeres es reconocida mundialmente como un grave problema de salud pública y una de las violaciones de los derechos humanos más sistemática y generalizada. La violencia de pareja se compone de acciones lesivas de naturaleza física, sexual, psicológica o financiera en cualquier tipo de unión romántica, incluyendo los comportamientos manipuladores^1^. Además de provocar daños físicos, la violencia de pareja se asocia con un mayor riesgo de presentar depresión y ansiedad, trastorno de estrés postraumático e intentos de suicidio^2^^,^^3^^,^^4^^,^^5^^,^^6^^,^^7^^,^^8^^,^^9^. La violencia de pareja debe situarse en relación con las múltiples formas de violencia que sufren distintas mujeres^10^, entendidas desde una perspectiva interseccional, asociadas con diversos sistemas de opresión que les atraviesan, entre ellos, racismo, clasismo, capacitismo^11^. Asimismo, debe entenderse en relación con la violencia estructural, consistente al daño que ocasionan en las personas las fuerzas político-económicas globales y locales, que derivan en acceso desigual a recursos, servicios, derechos y seguridad^12^^,^^13^. De esta manera, aunque la violencia de pareja trasciende las categorías étnicas, culturales y socioeconómicas, afecta a las mujeres de manera diferente según su contexto y sus recursos para hacerle frente^11^.

La Encuesta Nacional sobre la Dinámica de las Relaciones en los Hogares 2021 (ENDIREH) muestra que, en México, el 39,9% de las mujeres ha sufrido violencia de pareja en el transcurso de su vida y el 20,7% en los últimos 12 meses^14^. El estado de Chiapas, con el 51% de su población que vive en zonas rurales^15^, es la entidad federativa con la menor prevalencia reportada de violencia de pareja en el año anterior al levantamiento de esta encuesta (12,6%), con cifras más altas en contextos urbanos que en rurales^14^. Esta tendencia se corrobora también con los datos de las ediciones anteriores de la ENDIREH^16^. Sin embargo, Chiapas ocupa el lugar más bajo en el Índice de Desigualdad de Género de todo el país, según cálculos propios a partir de la metodología del Programa de las Naciones Unidas para el Desarrollo (PNUD)^17^, la cual está teóricamente asociada a la violencia de pareja^18^^,^^19^. Esta desigualdad de género adquiere matices particulares debido a otros sistemas de estratificación: 67,4% de la población vive en condiciones de pobreza, el 28,2% en pobreza extrema^20^ y el 28,2% de la población habla alguna lengua indígena^15^.

Aunque los bajos niveles de reporte de violencia de pareja en zonas rurales de Chiapas pueden atribuirse a aspectos metodológicos vinculados con la pertinencia cultural de los instrumentos de recolección de datos, su administración y lenguaje^21^^,^^22^, sorprende el caso de la comunidad estudiada en este artículo. En esta comunidad rural no indígena, altamente marginada, de aproximadamente 1.200 habitantes, ubicada en la región Frailesca del estado de Chiapas, se llevó a cabo en 2017 un estudio de métodos mixtos cuyo componente cuantitativo mostró niveles extremadamente elevados de violencia física y sexual en comparación con las cifras nacionales y estatales^4^. En la ENDIREH 2016, realizada un año antes de recabar los datos para este estudio, el porcentaje de mujeres en Chiapas que reportó haber padecido algún evento de violencia física o sexual por parte de su actual o última pareja fue del 16,1%^16^. En cambio, en esta comunidad rural no indígena, dos de cada tres mujeres (66,4%) admitieron haberla padecido^4^.

Teniendo en cuenta las múltiples consecuencias de la violencia de pareja sobre la salud física y mental^2^^,^^3^^,^^4^^,^^5^^,^^6^^,^^7^^,^^8^^,^^9^^,^^23^^,^^24^ es de relevancia estudiar las dinámicas y patrones de búsqueda de ayuda de mujeres afectadas por la violencia de pareja en contextos marginalizados, dado que, en México, los servicios de salud podrían tener un papel central tanto en la prevención como en la atención de la violencia de pareja, así como en referir a las mujeres a instancias de procuración de justicia, trabajo social, apoyo de la familia, etc., tal y como lo establece la Norma Oficial Mexicana sobre Violencia Familiar, Sexual y Contra las Mujeres, también conocida como la NOM-046^25^. 

### Estrategias de búsqueda de ayuda y dinámicas de violencia de pareja

La violencia de pareja no es homogénea. Algunas dinámicas emergen del deseo de dominación y control, mientras que otras son fruto de acumulación de tensiones que terminan en violencia. Es por eso que es preciso diferenciar entre dos dinámicas de violencia de pareja: el terrorismo íntimo y la violencia situacional^28^^,^^29^. La primera de estas dinámicas comprende un patrón general de conductas controladoras vinculadas al poder masculino, y se asocia con daños severos a la salud física y mental de las mujeres, incluyendo el feminicidio^30^^,^^31^. A nivel poblacional, el 1,2% de todas las mujeres mexicanas mayores de 15 años se encuentran en esta situación de terrorismo íntimo^30^. La segunda, la violencia situacional, es el resultado de tensiones y conflictos no asociados al control patriarcal y, a nivel nacional, el 5,8% de las mujeres se encuentra en esta situación^30^. 

En la población estudiada en este artículo, se han descrito previamente estas dinámicas, identificando cifras considerablemente más altas para ambos tipos de violencia que las reportadas a nivel nacional, con una prevalencia mayor del terrorismo íntimo (40,6%) que de la violencia situacional (25,8%)^4^. 

Las consecuencias para la salud también son más graves entre las que sufren terrorismo íntimo. En esta misma comunidad, el terrorismo íntimo está asociado con un riesgo 6,6 veces mayor de sufrir síntomas depresivos graves y el doble de riesgo de presentar ideación suicida^4^. Además, los patrones de búsqueda de ayuda son contingentes al tipo de violencia experimentada. Los datos de la ENDIREH 2016 muestran que el porcentaje de mujeres que pidió ayuda formal por situaciones de violencia de pareja, en instituciones públicas (incluyendo servicios de salud) es casi el triple en el caso de mujeres en situaciones de terrorismo íntimo que en dinámicas de violencia situacional de pareja (16,8% vs. 5,5%)^30^.

Las mujeres no son víctimas pasivas: desarrollan estrategias de afrontamiento al reconocer su vulnerabilidad y gestionar recursos simbólicos, materiales y relacionales^32^. Contarle a alguien la situación de violencia es una forma de buscar ayuda, ya que activamente puede estar buscando apoyo para poner fin a la violencia, así como reconocimiento, comprensión y acompañamiento emocional^32^^,^^33^. El proceso de búsqueda de ayuda puede ser formal (en instituciones públicas o comunitarias) o informal (con personas que conforman la red de apoyo).

La decisión de revelar la violencia se ve influida por el reconocimiento del acto violento y la posibilidad y deseo de buscar ayuda^34^. En esta decisión influyen aspectos individuales y estructurales. Por ejemplo, según cálculos propios a partir de la ENDIREH 2021^14^, el 4,6% de las mujeres mayores de 15 años que han sido golpeadas consideran estos eventos como de poca importancia; el porcentaje asciende al 7,2% entre las que fueron amarradas por su pareja. Además, en este proceso también interviene la evaluación de potenciales riesgos relacionados con sus recursos materiales, relacionales, económicos, sociales e institucionales^35^. Por ejemplo, en México, la atención en salud suele ser deficiente tras episodios de violencia^36^^,^^37^. Considerar que tras estos eventos de violencia la búsqueda de ayuda no era importante, puede implicar tanto que el evento en sí no se consideró relevante, como que la búsqueda de ayuda no se consideró necesaria, útil o prioritaria.

Aunque el abordaje tradicional para la violencia de pareja se centra en violencia física y sexual grave, y en promover la denuncia en instancias de procuración de justicia, seguridad pública o asistencia a víctimas, solo el 8,3% de las sobrevivientes buscan ayuda en instituciones públicas^30^. Esto puede ser aún menor en comunidades rurales debido a barreras geográficas y económicas, así como a particularidades en cómo se concibe y aborda la violencia de forma comunitaria.

Incluso teniendo acceso a instituciones gubernamentales, muchas mujeres no buscan ayuda formal por miedo a las represalias de su pareja, familiares o integrantes de la comunidad, miedo a no ser creídas, vergüenza, culpa y culpabilización por parte de la sociedad y personas prestadoras de servicios públicos, así como desconfianza en las instituciones^30^^,^^38^^,^^39^^,^^40^^,^^41^. Por lo tanto, las mujeres suelen buscar ayuda informal. Un estudio en 31 países del Sur Global mostró que el 34,9% de las mujeres en situación de violencia acuden principalmente a familiares, especialmente, en casos severos, si han visto violencia en su historia familiar o si tienen mayores recursos económicos^42^. En comunidades pequeñas, la pertenencia social también favorece la búsqueda de ayuda informal^43^. Aunque la ayuda informal no siempre pone fin a la violencia, puede aumentar el acceso a la información y mejorar el apoyo social y económico^35^^,^^44^.

Sin embargo, muchas mujeres temen revelar la violencia ante sus redes sociales, por falta de apoyo familiar o por la percepción de que la violencia es inevitable o justificada por los roles de género^40^^,^^45^^,^^46^. A nivel nacional, el 41% de las mujeres que denuncian en instancias públicas no se lo mencionaron a sus familias, principalmente, por mandatos culturales provenientes del *familismo* que reproducen estructuras patriarcales^46^. Esto cobra especial relevancia en comunidades pequeñas porque las redes sociales de apoyo son cruciales en casos de emergencia para acceder a dinero o transporte y podrían fracturarse al revelar la violencia ejercida por integrantes de dichas redes^47^. 

Sin duda, el caso de esta comunidad rural es lo que podríamos considerar un caso extremo^26^, el cual si bien no podemos generalizar^27^, nos puede arrojar datos relevantes sobre la búsqueda de ayuda, el tipo de ayuda requerida, así como los factores inhibidores para buscar ayuda formal o que impiden buscarla. Este estudio permite ilustrar factores sociales, culturales y estructurales que están relacionados con la búsqueda de ayuda, centrándonos en cómo las distintas dinámicas de violencia de pareja pueden impactar la decisión de buscar ayuda. Sobre este conocimiento, analizamos el papel que pueden desempeñar las intervenciones de salud comunitarias para facilitar la búsqueda de ayuda tanto formal como informal, así como para brindar atención y recursos para mejorar la salud de las sobrevivientes. Esto es particularmente relevante, ya que la literatura sobre la búsqueda de ayuda en las zonas rurales de México es limitada, por lo cual esta investigación puede fortalecer los servicios de salud rurales desde una perspectiva de género^25^.

## Métodos

El presente artículo parte de un estudio convergente de métodos mixtos que se realizó entre julio y diciembre de 2017, en una comunidad rural con aproximadamente 1.200 habitantes en la región Frailesca de Chiapas^47^. La comunidad se encuentra a cinco horas de la ciudad más cercana (en automóvil). La mayor parte de la población masculina se dedica al cultivo del café. La mayoría de la población profesa la religión católica, con algunos seguidores de las religiones presbiteriana y pentecostal. Las decisiones comunitarias se toman en las reuniones de la Asamblea Ejidal, el órgano de gobierno local conformado por los ejidatarios, es decir, los titulares de derechos sobre las parcelas de tierra que integran el ejido, una forma de tenencia colectiva de tierra, con uso individual o familiar, reconocida legalmente en México^36^*.* En esta comunidad, al momento del estudio, solo 5 de los 85 ejidatarios eran mujeres. En estas asambleas se elige anualmente a un pequeño grupo de autoridades locales (presidente, tesorero y secretario del comisariado ejidal, el agente rural, el juez rural y el consejo de vigilancia) para desempeñar funciones de salvaguarda en la comunidad. En conjunto, estas autoridades se encargan de la administración del ejido, aseguran que se respeten los acuerdos y el orden público, resuelven conflictos agrarios, representan políticamente al ejido e informan a las autoridades municipales sobre irregularidades^36^.

En esta comunidad trabaja la organización no gubernamental Compañeros en Salud (CES), que trabaja con la Secretaría de Salud desde 2011, brindando atención de salud en diez clínicas rurales, un hospital y dos casas maternas. El estudio surgió después de que el personal de salud identificara síntomas depresivos como una de las principales causas de consulta en las mujeres^48^, y la violencia de pareja como uno de los principales factores desencadenantes^49^^,^^50^.

Se realizó una encuesta con muestreo probabilístico aleatorio simple entre julio y agosto de 2017. Se calculó una muestra de 159 mujeres de 15 años o más, corrigiendo por población finita. Para la selección de la muestra, se enumeraron y eligieron de manera aleatoria las casas participantes a partir de una lista obtenida del centro de salud, una vez elegidas las casas, se enumeraron a las mujeres que cumplieran con los criterios de inclusión dentro de cada casa y nuevamente se eligieron de manera aleatoria. Los criterios de inclusión fueron: 1) ser mujer, 2) tener al menos 15 años, 3) ser residente actual de la comunidad; y los criterios de exclusión: 1) tener una discapacidad auditiva, cognitiva o problemas de lenguaje, y 2) que alguna otra persona que viva en su casa participara en entrevistas o encuestas de este estudio (para asegurar confidencialidad). Se obtuvo una tasa de respuesta del 89% y el relevamiento corrió a cargo de tres de las autoras y una asistente de investigación. 

Las preguntas relacionadas con la violencia de pareja y búsqueda de ayuda se adaptaron de la ENDIREH. Se categorizó la dinámica de violencia de pareja como *terrorismo íntimo* cuando se reportó alto control por parte de la pareja (más de cuatro conductas de control), violencia física y/o sexual independientemente de la severidad, y cuando se reportó control moderado (entre una y cuatro conductas de control) y violencia física y/o sexual de alta severidad. La *violencia situacional*, por su parte, se consideró cuando había violencia física y/o sexual sin conductas de control, o cuando el control era moderado y la violencia física y/o sexual era de baja gravedad^4^. Se categorizó la búsqueda de ayuda como *positiva* cuando las participantes refirieron haber hablado con alguien sobre la violencia que vivían, si habían buscado ayuda con las autoridades locales civiles, religiosas y de salud o si buscaron ayuda con abogados o en instancias gubernamentales. Se registró a quién habían pedido ayuda las participantes y la respuesta que obtuvieron de estas personas. Para los resultados cuantitativos presentados en el presente artículo se utilizó el programa Stata 15 y se realizó un análisis descriptivo reportando porcentajes para los resultados de variables categóricas.

Adicionalmente, y con el objetivo de profundizar y contextualizar los datos cuantitativos, dos de las autoras realizaron entrevistas en profundidad a diez mujeres (Tabla 1) y nueve informantes claves. Las mujeres fueron seleccionadas por la variabilidad de sus experiencias de violencia de pareja identificadas a partir de la encuesta. Las personas informantes claves, seleccionadas intencionalmente en función de su papel comunitario, fueron cinco autoridades religiosas (dos hombres y una mujer ministros católicos, un pastor pentecostal y un pastor presbiteriano) y cuatro autoridades locales civiles (tres hombres: presidente del comisariado ejidal, agente rural y representante del comité de vigilancia; y una mujer: síndico). Durante el análisis de datos se identificó que tanto la mujer ministra como la mujer síndico refirieron no haber recibido solicitudes de apoyo relacionados con violencia de pareja, por lo que los testimonios presentados son únicamente de los siete participantes hombres. 

**Tabla 1:** Características de las mujeres que participaron en las entrevistas en profundidad. Región Frailesca de Chiapas, México, 2017.

Seudónimos	Edad	Años de escolaridad	Violencia de pareja
Eréndira*	34	1	Situacional. Testigo de terrorismo íntimo en la infancia.
Marisol	36	12	Situacional
Yusmi	30	2	Situacional. Testigo de terrorismo íntimo en la infancia.
Dulce	20	11	No
Juana	26	9	Situacional
Rosa	39	9	Terrorismo íntimo
Romelia	25	9	No
Elba	27	4	Situacional
Flori	31	5	Terrorismo íntimo con pareja previa, violencia situacional con pareja actual
Guadalupe	37	4	Situacional

*Los nombres que aparecen en la tabla y a largo del artículo son seudónimos. Se presentan sólo datos de las mujeres entrevistadas, ya que autoridades locales y religiosas, podrían ser fácilmente identificadas por su cargo y edad.

Las entrevistas en profundidad y la observación participante se llevaron a cabo de julio a diciembre 2017 y exploraron las normas y los roles de género, las experiencias de violencia de género, la búsqueda de ayuda, y la respuesta a la violencia por parte de la mujer, la familia, las personas cercanas y la comunidad. Las entrevistas se realizaron en un espacio privado y duraron entre 45 minutos y dos horas. Las entrevistas fueron grabadas, transcritas y analizadas en español. La primera autora realizó observación participante dentro de la comunidad en diversos grupos y entornos sociales (por ejemplo, convivencia familiar, grupos religiosos, torneos deportivos, graduaciones, reuniones de Alcohólicos Anónimos).

Se realizó un análisis inductivo con enfoque temático desde una perspectiva de género, en el que las dos primeras autoras identificaron códigos en las partes de las entrevistas relacionadas con externar la situación de la violencia, la búsqueda de ayuda y la respuesta a estas. Posteriormente, se crearon categorías temáticas mediante un proceso iterativo de reflexión, volviendo a las transcripciones para aclaración y contextualización. Los conflictos sobre la formación tanto de códigos como de categorías se resolvieron por consenso. Las notas etnográficas de la observación participativa se centraron en las normas, creencias y expectativas de género en relación con la violencia de género y la búsqueda de ayuda, e informaron la contextualización de los resultados cualitativos. Durante el proceso iterativo del análisis cualitativo se identificaron cuatro temas que informaron la contextualización de los resultados cuantitativos: 1) limitación del daño, 2) desesperanza ante la impunidad, 3) normas y expectativas de género en conflicto, y 4) resistencia a la violencia. Se tomaron notas etnográficas de la observación participativa para contextualizar los resultados.

Se identificaron áreas de divergencia y convergencia de los datos cuantitativos y cualitativos. Los resultados cuantitativos y cualitativos se presentan en una narrativa integrada, estructurada a lo largo de las cuatro categorías temáticas derivadas del análisis cualitativo. Los resultados cuantitativos demuestran el alcance de las experiencias, mientras que los cualitativos ilustran el contexto dinámico de los resultados cuantitativos, describiendo dónde pueden encontrarse oportunidades para las intervenciones sociales y de salud. Los nombres reales se reemplazaron por seudónimos para proteger la identidad de las personas participantes. 

Este estudio se apegó a las directrices de la Organización Mundial de la Salud sobre consideraciones éticas y de seguridad para la investigación en violencia contra las mujeres^51^. La aprobación ética fue proporcionada por el Comité de Ética de la Oficina de Administración de Investigación Humana de la Escuela de Medicina de Harvard (IRB17-0583) y la Secretaría de Salud de Chiapas. Se obtuvo el consentimiento informado de las personas adultas y el asentimiento informado de las adolescentes participantes, tanto para la encuesta como para las entrevistas.

## Resultados

Se realizó un análisis descriptivo de las respuestas de las 141 mujeres que respondieron el cuestionario. De estas, el 76,6% estaban casadas o en unión libre, el 5% separadas o divorciadas y menos del 10% solteras, casi un tercio se unió a los 16 años o antes y el 90,8% tenía hijos. No terminó la primaria el 46,1%, menos de la mitad tenían recursos financieros propios y solo el 22% disponía de dinero en efectivo para una emergencia. Solo la mitad de las mujeres tenían a alguien de confianza para hablar de sus problemas. De las 141 mujeres, el 66,4% declaró haber sufrido violencia de pareja con la actual o última pareja, el 40,6% terrorismo íntimo y el 25,8% violencia situacional.

Los resultados de esta encuesta muestran que cuatro de cada diez mujeres de esta comunidad vivieron sus experiencias de violencia en silencio. La Tabla 2 muestra que el 58,8% de las mujeres que declararon haber sufrido violencia de pareja se lo contaron a alguien. Solo el 7,1% (todas sobrevivientes de terrorismo íntimo) buscó ayuda en instituciones o ante líderes comunitarios. La mayoría de las mujeres buscaron ayuda informal, en ambos casos en la familia de procreación; más sobrevivientes de terrorismo íntimo buscaron ayuda de su familia política (21,2%) que quienes se encontraban en violencia situacional (9,1%). Destaca el hecho de que las mujeres en dinámicas de violencia situacional no buscaron ayuda formal. Es importante destacar que compartir la experiencia con alguna persona no siempre acaba teniendo un impacto positivo en las mujeres.

**Tabla 2:** Búsqueda de ayuda formal e informal (en porcentajes), por tipo de dinámica de violencia, entre encuestadas que reportaron haber experimentado violencia de pareja. Región Frailesca de Chiapas, México, 2017.

Variables	Cualquier violencia n=85		Terrorismo íntimo n= 52		Violencia situacional n=33	
	n	%	n	%	n	%
**Lo comentó con alguien**						
No	35	41,2	21	40,4	14	57,6
Sí	50	58,8	31	59,6	19	57,6
**Ayuda formal (institucional o comunitaria)**						
No	79	92,9	46	88,5	0	0,0
Sí	6	7,1	6	11,5	0	0,0
Tipo de ayuda formal (institucional o comunitaria)*						
*Instituciones gubernamentales^a^*	2	2,4	2	3,8	0	0,0
*Autoridades locales^b^*	2	2,4	2	3,8	0	0,0
*Autoridades religiosas^c^*	0	0,0	0	0,0	0	0,0
*Personal de salud^d^*	1	1,2	1	1,9	0	0,0
*Otros (abogada)*	1	1,2	1	1,9	0	0,0
**Ayuda informal**						
No	36	42,4	21	40,4	15	45,5
Sí	49	64,6	31	59,6	18	54,5
Tipo de ayuda informal*						
*Familia de ella*	38	44,7	24	46,2	14	42,5
*Familia política*	14	16,5	11	21,2	3	9,1
*Amistades*	6	7,1	4	7,7	2	6,1

Fuente: Elaboración propia. *Respuestas múltiples. ^a^Ministerio Público, juzgado municipal, juzgado de paz y conciliación, presidencia municipal, sistema para el Desarrollo Integral de la Familia (DIF). ^b^Presidente del comisariado ejidal, agente rural, juez rural, secretario rural, ejidatarios adscritos como policías. ^c^Ministros o pastores de la comunidad. ^d^Personal de medicina o enfermería de la clínica comunitaria o de fuera de la comunidad.

### Limitación del daño

La mayor parte de las mujeres encuestadas que pidió ayuda informal se sintió apoyada, entre ellas el 78,9% de quienes buscaron ayuda con su familia, el 71,4% de quienes buscaron ayuda en su familia política y el 66,7% de quienes buscaron ayuda con amistades. El apoyo que mencionaron buscaba la limitación del daño y se resume en cuatro categorías identificadas a partir de las entrevistas: 1) apoyo emocional, 2) salvaguarda de la mujer, 3) intervención con el agresor, y 4) intervención de los hijos e hijas. Estas dinámicas de apoyo son atravesadas por los roles de género de los actores involucrados: por lo general, los hombres brindan apoyo desde una posición de autoridad frente a los agresores o la mujer, mientras que las mujeres brindan cuidado a las sobrevivientes e infancias. La falta de apoyo consiste en no reconocer o minimizar la violencia y recibir agresiones o culpabilización al externarla.

#### Apoyo emocional

El apoyo emocional es descrito por las participantes como uno de los más significativos, refiriendo alivio al ser escuchadas o aconsejadas, principalmente por parte de otras mujeres de la red familiar, independientemente del contenido del consejo o de su voluntad y capacidad de ponerlo en práctica. Sobrevivientes de violencia situacional describen mantener conversaciones reflexivas con personas cercanas, mientras que sobrevivientes de terrorismo íntimo relatan más recibir comentarios por parte de personas (familiares o no) que atestiguan la violencia.

*Mi hermana pues solo me escuchaba, lloraba conmigo. A veces me decía “déjalo, sepárate de él, no sé, a lo mejor vas a hacer una mejor vida” o sea cosas así. Y otros [cuñado] me decían “no lo dejes”* [...] *Ahora sí que pues la que tenía que tomar su decisión era yo* [...] *Y así pero sí, el apoyo de mi familia ahora sí que me ha ayudado mucho para salir adelante* (Marisol, 36 años, violencia situacional)

*“ay por qué lo aguantás a ese viejo, tanto no te pudistes conseguir otro más joven, déjalo, andá a cuidar tu familia”* [...] *Mis vecinos, a veces veían cómo me pegaba y eso es lo que me aconsejaban ellos, pero yo decía: ¿y mis hijos?* (Flori, 31 años, sobre experiencia de terrorismo íntimo)

#### Salvaguarda de la mujer

Las mujeres comúnmente brindan apoyo protegiendo a la mujer, otorgando un espacio seguro donde quedarse cuando su esposo la “corre” de la casa, lo cual significa que la mujer huye con miedo de su casa, normalmente con sus hijos e hijas, para evitar la violencia física. Generalmente las mujeres “se corren” cuando saben que su marido ha estado bebiendo alcohol, y que suele ser violento cuando bebe. Estos relatos suelen venir de mujeres que han vivido terrorismo íntimo o de mujeres que relatan la violencia que atestiguaron hacia sus madres. Como dice Yusmi de 30 años que vive violencia situacional y atestiguó terrorismo íntimo en la infancia “*si ella* [mi mamá] *no se corría, pues la mataba mi papá*”. Otras mujeres hablan de “*correrse*” incluso cuando no han sufrido violencia física, debido al conocimiento transgeneracional de que los hombres son violentos cuando beben.

*…nosotros a veces íbamos a quedar en la casa de una mi tía, pero como ella, el esposo no toma, nosotros llegábamos y no llevábamos cobija ni nada, nada más prestaba mi mamá un costal y lo tiraba en el piso.* (Eréndida, 34 años, violencia situacional, testigo de terrorismo íntimo contra su madre en su infancia)

#### Intervención con el agresor

Al buscar ayuda informal, distintas personas suelen interponerse entre el agresor y la mujer, con mayor frecuencia familiares hombres, ya que las mujeres pueden correr peligro. En una entrevista incluso se menciona que cuando su suegra se interpuso entre su esposo y ella, su esposo también la golpeó. Los hombres suelen amenazar al agresor, corregirlo o contenerlo físicamente. La contención física es descrita en las entrevistas solo por sobrevivientes y testigas de terrorismo íntimo. De acuerdo con las encuestas (Tabla 2), las sobrevivientes de terrorismo íntimo buscan ayuda de familiares del agresor (21,2%) en mayor medida que sobrevivientes de violencia situacional (9,1%). Este tipo de apoyo busca reducir el daño en el momento de la violencia física y disminuir o detener la violencia en el futuro, aunque esto último rara vez sucede.

*Yo no le hice algo para que él, o sea para que él reaccionara así ¿no?* […] *No, solito se enojó, quién sabe* […] *no sé si ha escuchado a engazarse, que como a enojarse, así que se le suben bastante sus nervios, entonces empezaba a tirar todas las cosas que encontrara ahí, entonces hasta que lo amarraran de las manos y de los pies entonces ya se dormía*. (Rosa, 39 años, terrorismo íntimo)

*…le decían, o sea a él, pero ya no, no hacía caso.* […] *Bueno el papá le decía que no fuera así, que por eso son esposos para que se lleven bien, no para que estén peleando y tú la estés golpeando.* (Rosa, 39 años, terrorismo íntimo)

[mi papá] *me dijo “mira hija, yo no voy a ir a su casa porque yo no fui a entregarte ahí, ve y dile que venga aquí, yo voy a arreglar las cosas* […] *mira, si no quieres a mi hija, está bien, tal vez se terminó ese amor, pero háblale claro, tampoco la estés lastimando”.* (Juana, 26 años, violencia situacional)

#### Intervención de los hijos

Los hijos y las hijas con frecuencia brindan apoyo instrumental, sobre todo en el contexto de terrorismo íntimo. No tienen roles marcados por el género y proveen los tres tipos de apoyo mencionados anteriormente. Entre las estrategias a las que recurren destacan pedir a su padre que se detenga, distraerlo, o facilitar que su madre salga de la casa. También contienen físicamente al agresor (arriesgándose), piden ayuda y brindan apoyo emocional a su madre. Varias participantes entrevistadas consideran que la intervención de sus hijas e hijos les ha salvado la vida. Cabe mencionar que todas las entrevistadas que recordaron atestiguar la violencia de parte de su padre hacia su madre lo contaron con rechazo hacia esta y con miedo.

*Y una vez mi hermana sí la defendió, pero mi papá lo dio [a ella] una manada en la boca del estómago y lo tiró sobre el molino, y así se quedó, y ella no volvía. Y ya en lo que nosotros fuimos a llamar a mi tío ya él llegó, ya mi papá salió a pegarle a mi tío. Ya fue en ese momento que mi mamá logró escaparse, a correrse ella con todos nosotros pues*. (Eréndida, 34 años, violencia situacional, testigo de terrorismo íntimo contra su madre en su infancia)

*Entonces yo le dije a la niña que me ayudara, entonces y la niña fue que le dijo “papi, soltá a mi mami porque no lo vayas a matar” o algo así. “Solo por ti lo voy a hacer, lo voy a soltar a tu mamá porque yo ya la odio y ya no la quiero ver aquí en la casa, pero solo porque me lo estás pidiendo tú, lo voy a dejar”.* (Rosa, 39 años, terrorismo íntimo)

#### Falta de apoyo

Las mujeres entrevistadas relataron con dolor situaciones en las que no solo no fueron apoyadas por sus familiares, sino que recibieron maltrato emocional. De acuerdo con las encuestas, el 21,1% de las mujeres que comentaron la violencia con sus propios familiares y el 28,6% de las que buscaron ayuda con su familia política no se sintieron apoyadas. Entre ellas, algunas refirieron recibir maltrato refiriéndose específicamente a que las ignoraron (10,3% propia familia, ninguna lo reportó de la familia política), o las humillaron, no les creyeron o las culpabilizaron (2,6% propia familia, 14,2% familia política) por la violencia vivida. 

Además, comentar la violencia a la familia se obstaculizaba al temer la reacción de los padres, ya sea por la experiencia previa de violencia física o verbal parental hacia ellas, frecuente entre las mujeres entrevistadas, o por la desaprobación de los padres sobre la unión.

*Una vez ya me había dejado bien lastimada aquí, y incluso vino mi suegra. Y mi suegra no vino a defenderme, sino que vino a echarle más leña el fuego, vino a acabarme a mí y a defenderle a él* [esposo] […] *Luego me vine con mi mamá y mi mamá me gritaba como siempre, decía yo “no es vida para mí porque salgo de las brasas, vengo a caer en las llamas”. No había paz en ninguna parte.* (Eréndida, 34 años, violencia situacional, testigo de terrorismo íntimo en la infancia)

*En una ocasión me dejé con mi esposo ya le empecé a platicar* [a mi mamá] […] *y me dice “tal vez no es verdad lo que andas contando”* […] *“ay y cuándo te visto que andes golpeada”.* (Juana, 26 años, violencia situacional)

*Yo me embaracé como al año de mi primer niño, empezaba a tomar* [mi esposo] *y llegaba yo con mi papá y me acercaba yo bien triste con él y me decía “mira, te dije, para que no entendías, te dije, mira”.* (Elba, 27 años, violencia situacional)

### Desesperanza ante la impunidad

El bajo índice de búsqueda de ayuda institucional que se aprecia en la Tabla 2 puede explicarse en parte por barreras socioeconómicas y geográficas que limitan la capacidad de las mujeres para llegar a ellos. La comunidad se encuentra de una a tres horas en automóvil de las oficinas del ministerio público. No hay señal telefónica y el acceso a Internet es limitado, costoso y solo disponible en espacios públicos. La mitad de las mujeres encuestadas dependen económicamente de sus maridos y/o suegros, y menos de la mitad tienen a alguien que cuide de sus hijos e hijas si tienen que salir de la comunidad**.** Además, no es común que se cumplan los acuerdos o que cese la violencia si interponen denuncia. Entre las mujeres que participaron en la encuesta, dos buscaron apoyo en las instituciones gubernamentales de justicia, en un caso al denunciar se multó a la pareja, y en otro se llegó a un acuerdo conciliatorio. El siguiente testimonio ilustra el resultado de un acuerdo:

*En ese tiempo yo le puse una demanda* [a mi esposo]. […] *Pues llegó hasta juzgado de paz* […] *lo que hice fue poner la demanda porque me había golpeado y todo. Llegamos a un acuerdo que él pasaría la pensión y todo eso, cosa que nunca cumplió. Y así, pero sí... me golpeó.* (Marisol, 36 años, violencia situacional)

Por otro lado, pedir ayuda a las autoridades locales tampoco asegura la salvaguarda de las mujeres. Las consecuencias acordadas incluyen una multa que se paga a la Asamblea Ejidal (que se puede perdonar) y puede empobrecer más a las familias, acuerdos conciliatorios entre la pareja, encarcelamiento temporal en la comunidad (normalmente menos de 24 horas) o emisión de un acta de hechos. Por lo tanto, no sorprende que tan pocas mujeres recurran a ellos (Tabla 2).

*Bueno aquí, depende como a veces en la comunidad, depende la asamblea* […] *Ellos ponen una multa de, de 500 pesos. ¿Sí? A, a los que tomen pue.* […] *Mucha gente a veces lo ven duro la multa.* […] *ni aún a veces nosotros no, como autoridad a veces no, no aplicamos la multa como debe ser.* […] *a veces como humanos que somos, pues creo que, la amistad nunca debe acabar entre, como, como hombres*. (Autoridad religiosa que fungió previamente como autoridad civil)

Flori, de 31 años que vivió terrorismo íntimo con su pareja previa ilustra su experiencia después de realizar un “acuerdo” en la comunidad sobre cuánto tiempo podía su ex pareja llevarse a sus hijos:

*Se los llevó por tres días, y ya de esos tres días ya va a hacer dos años* […] *Tocamos autoridad aquí, pero cuando él estaba todavía y los niños estaban aquí y le dijeron que* [se los podía llevar] *mínimo con permiso mío y máximo 15 días, más ya no. Pero le valió.* (Flori, 31 años, terrorismo íntimo)

Además, las autoridades locales han sido históricamente hombres. La única autoridad local entrevistada, que era mujer, refirió obtener el cargo sin desearlo después de que su esposo fuera elegido por la asamblea ejidal debido a que “*cambió la regla, que tenía que ser mujer*”. Ella misma refirió no participar en la asamblea porque “*eso es aparte, más de hombres*”, ilustrando que desconoce que su cargo le confiere voz y voto en esta. De acuerdo con los testimonios de los hombres que fungen como autoridades locales, solo suelen intervenir cuando la violencia causa escándalo público o cuando está asociada con el consumo de alcohol.

*Y pues ya fue que en junio me hizo ese escándalo, lo llevaron [las autoridades locales] a la cárcel [...]. Él sabía que borracho no podía entrar en la casa, no, porque yo conocía su reacción y no, no me gustaba [...]. Y ese día llegó y quería entrar y le dije que no, que no podía entrar y empieza a hacer su escándalo que yo tenía querido y que no sé qué cosa, a patear la puerta, a forzarla [...] y la abrió, la dejó bien golpeada la puerta. (Marisol, 36 años, violencia situacional)*

*Entrevistadora: ¿Qué pasa cuando hay una mujer que es golpeada por su esposo, aunque no esté tomado?*

*Autoridad local: No, ahí sí se llama, es un maltrato, como se dice, directamente a la persona y eso nosotros lo turnamos al ministerio público porque ya el ministerio le tendrá que aplicar la ley.*

Asimismo, las autoridades responden minimizando la violencia, justificándola y reforzando que la violencia de pareja se debe atender en el ámbito privado, desincentivando la búsqueda de ayuda. Llama la atención que las autoridades entrevistadas refieren que no les ha tocado atender casos “graves” de violencia de pareja, mientras que en los datos de la encuesta podemos observar que solo mujeres sobrevivientes de terrorismo íntimo buscaron ayuda con las autoridades locales (Tabla 2).

*...tiene que haber alguna justificación por qué la persona regañó, se enojó con su esposa o algo, pero tiene que aceptarlo cuando uno cometió el error. Hay personas que sí lo aceptan, piden disculpa, pero eso es en familia pues, ya no es en autoridad. Algún otro problema grave aquí no he visto, nada más problemas o maltratos o… verbales donde de repente viene borracho, golpea la puerta, corre a su esposa, pero eso es verbal, no les pega.* (Autoridad local)

Como se expuso antes, “correr a su esposa” o “correrse de su casa” implica que la mujer tiene miedo a la violencia física severa. Esta minimización y condonación de la violencia, junto con el deseo de mantener la unión familiar (reforzado por valores cristianos), fomenta que los esfuerzos de las autoridades locales (civiles y religiosas), se enfoquen en facilitar procesos de perdón y conciliación para evitar la separación de la pareja:

*Una pareja, que ya estaban casi, a punto de, de terminar ya, su esposo los corría de la casa, y se espantaban. Nos acercamos, sí aceptaron la plática… Y este, y al final, se perdonaron.* (Autoridad religiosa)

*Hay parejas que se pelean, le pegan a sus esposas borrachos, los demandan y las señoras que llegan a la agencia y mencionando de que pues…. que ya no quieren estar junto con el esposo porque se porta mal y todo eso. Se hace un acta, por acuerdo de la pareja, de la esposa, decide ya no vivir con la persona, pero eso uno de hombre “no, dame una oportunidad” hacen acuerdo, pero quieren el papel de que si lo vuelve a hacer por segunda vez, ya la mujer pues se va a su casa* [se separa*].* (Autoridad local)

### Normas y expectativas de género en conflicto

En cuanto a las razones para no buscar ayuda capturadas en la encuesta, la Tabla 3 muestra que las principales fueron: 1) para que no se enteren otras personas (28,6%), cifra mayor entre las mujeres en dinámicas de terrorismo íntimo que en violencia situacional (respectivamente, 33,3% y 21,4%), 2) vergüenza (22,9%), y 3) miedo (20%). Asimismo, el 23,8% de las mujeres que sufrieron terrorismo íntimo mencionaron que su familia las convenció para que no lo revelaran a otras personas o autoridades, a diferencia de ninguna que vivió violencia situacional. Cuatro mujeres (28,6%) que vivieron violencia situacional no buscaron ayuda “porque no era importante”, frente a solo una mujer que experimentó terrorismo íntimo. Otra diferencia significativa, es que, entre las sobrevivientes de terrorismo íntimo, el 23,8% no reveló la violencia por temor a que la maltrataran, culparan o no le creyeran.

**Tabla 3:** Razones para no pedir ayuda entre encuestadas que reportaron haber experimentado violencia de pareja, por dinámica de violencia. Región Frailesca de Chiapas, México, 2017.

Razones	Cualquier violencia de pareja (n=35)		Terrorismo íntimo (n=21)		Violencia situacional (n=14)	
	n	%	n	%	n	%
Para que otros no se enteraran	10	28,6	7	33,3	3	21,4
Vergüenza	8	22,9	5	23,8	3	21,4
Miedo	7	20,0	3	14,3	4	28,6
Su familia la convenció de que no lo hiciera	5	14,3	5	23,8	0	0,0
No era importante	5	14,3	1	4,8	4	28,6
Otros (su madre la trataba mal/la culpaba/le pegaba, no la creían)	5	14,3	5	23,8	0	0,0
No habría hecho ninguna diferencia / no le creerían	3	8,6	2	9,5	1	7,1
Amenazas	1	8,9	1	4,8	0	0,0
Por los hijos	1	2,9	1	4,8	0	0,0
Esa persona tenía derecho a hacerlo o ella lo merecía	1	2,9	1	4,8	0	0,0
Porque dicen que hay que aguantar	1	2,9	1	4,8	0	0,0
No sabía que podía pedir ayuda	0	0,0	0	0,0	0	0,0
Necesitaba dinero y no tenía	0	0,0	0	0,0	0	0,0

Fuente: Elaboración propia.

En las entrevistas se mostró claramente que la decisión de buscar ayuda está influida por normas de género. Las autoridades hablan de que las mujeres causan la violencia al incumplir mandatos de género como no realizar tareas domésticas, no “*atender*” a sus maridos y no comportarse apaciblemente. Las mujeres entrevistadas mencionan escuchar este discurso también entre familiares, en su mayoría hombres. El siguiente testimonio de una autoridad local describe cuándo cree que las mujeres “*tienen la culpa*” de recibir golpes:

*...en que uno va al campo y llegamos y no tiene la comida. Hay todo eso, pues se tiene que molestar alguien y el hombre viene con hambre y no hay nada. A veces empiezan los problemas así…* (Autoridad local)

Inicialmente, en las encuestas, varias mujeres negaron haber experimentado violencia de pareja, sin embargo, la admitieron cuando se preguntaba si su pareja había sido violento cuando había tomado alcohol. El tomar alcohol es parte de la masculinidad hegemónica y es percibido como algo intrínseco al *ser hombre*^52^. En la comunidad, la violencia asociada al consumo del alcohol es más tolerada porque se considera una consecuencia del alcohol y no como acciones intencionales más fácilmente reconocibles como violencia^47^. La falta de reconocimiento de la violencia, cuando sucede en el contexto de intoxicación etílica, también se observa en el discurso de las autoridades con la creencia de que, bajo su efecto, los hombres no pueden controlarse. Incluso, como forma de prevenir enfrentamientos violentos, la Asamblea Ejidal prohibió la venta de alcohol dentro de la comunidad.

*...el borracho, por ejemplo, vino, se perdió y se pasó de copas, no supo qué dijo, se le perdona* [la violencia] *porque está alcohólico más que nada. Pero el que está de sano juicio hay que checarlo si no está mal de la mente con un doctor porque dice cosas que no debe decir.* (Autoridad local)

Al mismo tiempo, estas normas tradicionales de género se han debilitado con el tiempo y se dice repetitivamente que las mujeres de hoy en día “no deben soportar la violencia”. Como consecuencia, sufrirla puede ser motivo de vergüenza. El conflicto entre reconocerla, la expectativa de “no dejarse” y no poder detenerla, aunado a las enseñanzas religiosas y relativas a los mandatos de género, genera ambivalencia entre algunas mujeres.

*Ahí sí tengo una duda. Porque según dicen los psicólogos y todo eso, que si una mujer no quiere* [tener relaciones sexuales]*, su marido no la obligue, pero yo voy a la iglesia, soy católica entonces nos dice el padre que es una obligación como mujer entonces le digo ¿cuál está bien y cuál está mal?* (Juana, 26 años, violencia situacional)

Asimismo, existe una escasez de trabajos remunerados para las mujeres en la comunidad, limitándose a pequeños negocios insuficientes para cubrir gastos esenciales. Además, los roles de género restringen la participación de las mujeres en el campo, también limitada por labores de crianza y de reproducción doméstica. Esta situación económica limita las posibilidades de las mujeres de buscar ayuda formal, presentar una denuncia, o separarse.

*Uno de mujer no puede uno salir a trabajar* […] *Pues el marido no nos deja. Y o sea otro poco que no hay en qué. Nada más quedarnos en la casa, hacer los quehaceres de la casa. Ya el marido se encarga de trabajar y traer lo que vamos a necesitar.* (Flori, 31 años, violencia situacional en relación actual, terrorismo íntimo en relación anterior)

Sin embargo, la siguiente entrevistada, la única con un empleo formal, describe que cuando la economía no es un impedimento para la separación pesa más el deseo de tener una familia unida. Cabe mencionar que muchas entrevistadas que crecieron sin un padre añoraban que estos estuvieran presentes para sus hijos e hijas.

*…o sea eran tantas cosas, decía yo cómo, a mí no me preocupaba tanto la parte económica, cómo solventar mis gastos. Me preocupaba lo moral quizás, cómo va a vivir mi hijo sin cariño, sin amor de padre, sin apoyo…* (Marisol, 36 años, violencia situacional)

Por último, las mismas normas de género relegan a las mujeres a sus hogares, limitando su socialización fuera de la familia. En la comunidad, los espacios públicos están dominados por los hombres, y las salidas de mujeres sin una tarea específica están mal vistas. Los comportamientos controladores del terrorismo íntimo aíslan aún más a las mujeres, quienes adaptan su comportamiento, buscando evitar la violencia física. Muchas mujeres mencionaron a lo largo del estudio no tener amistades y de las encuestadas solo la mitad refirió tener a quien contarle sus problemas y, por lo tanto, no sorprende que solo el 7,1% de las mujeres que vivieron cualquier tipo de violencia la contaran con amistades (Tabla 2).

*Cuando éramos novios, él siempre me vigilaba. Siempre que no podía salir o no me dejaba tener amigos. Y tal vez por lo mismo que no me gusta salir porque no, ya me acostumbré de no, de estar encerrada ahí, de no salir.* (Rosa, 39 años, terrorismo íntimo)

*Bueno, yo al menos aquí como no salgo no tengo confianza o conocer con quién confiar, entonces este, o sea guardarlo pues. A veces yo me siento triste o a veces me siento contenta, no sé, ni entiendo por qué, pero quisiera compartirlo con alguien, pero no. A veces yo con mi marido “tú mira esto me pasó, o quiero esto o mirá esto” pero no me toma en cuenta.* (Flori, 31 años, terrorismo íntimo en una pareja anterior, violencia situacional con pareja actual)

Este último testimonio ilustra el deseo de las mujeres de compartir sus sentimientos. Algunas mencionaron después de las encuestas que querían hacerlo con psicólogas (que no hay en la comunidad) o doctoras. Sin embargo, Flori, una de las entrevistadas, expresó que, aunque consideró contar su historia con la doctora de la clínica, no pudo hacerlo: “*da pena* [angustia] *contar nuestra historia*”. Durante la recolección de datos, muchas mujeres agradecieron el espacio para ser escuchadas y afirmaron sentirse aliviadas al poder decir cosas de las que no habían podido “*desahogar*”.

### La resistencia ante la violencia

Finalmente, si las mujeres no buscan ayuda, no significa que no actúen frente a ella o que no la identifiquen. Las mujeres que relatan experiencias de violencia situacional buscan establecer límites con sus parejas (y familiares) a través del diálogo reflexivo, las amenazas o la fuerza física, buscando romper con los mandatos tradicionales de género (incluso internalizados) y la violencia transgeneracional, con el objetivo de construir para ellas y sus hijos e hijas una vida diferente a la que vivieron con sus padres, como dice Flori “*una buena vida llena de tranquilidad, paz y amor*”. En los discursos, hablan de que no quieren “*aguantar*” como sus madres, de que son capaces de sacar adelante a sus hijos solas (sobre todo si trabajaron previamente en la ciudad), refieren haber mencionado a sus parejas la posibilidad de separación, y platican con sus parejas de cómo sus experiencias previas influyen actualmente en su conducta.

*…me agarré una idea de que si me pega le pego. O sea, ya no soporto por lo mismo, mis nervios, ya no soporto que me va a pegar y me voy a quedar con los brazos cruzados, no.* (Flori, 31 años, violencia situacional en relación actual, terrorismo íntimo en relación anterior)

*A veces, pienso y digo, pues yo lo provoqué, estaba borracho, y pues que yo lo provoqué. Pero luego digo no, él no tiene ningún derecho de golpearme.* (Marisol, 36 años, violencia situacional)

[Le dije a mi esposo] *tienes que estar bien tú primero en la casa como cabeza, porque un día mis hijos van a crecer, van a mirar que esté pegando a su mujer, qué les vas a decir, con qué derecho* […] *porque tú también tomás y sos violento. Antes de que mis hijos crezcan tienes que cambiar* […] *Porque da vergüenza, la gente se da cuenta.* (Eréndida, 34 años, violencia situacional)

Por otro lado, las mujeres sobrevivientes de terrorismo íntimo entrevistadas evitan la provocación del agresor, incluso ante humillaciones, insultos y amenazas constantes, producto del miedo a la violencia severa por la que se han estado cerca de morir. Estas mujeres expresan sentirse solas y relatan encerrarse a llorar porque “*otra cosa no podía hacer*”. 

## Discusión

Este estudio muestra que el porcentaje de mujeres que buscan ayuda formal o informal ante situaciones de violencia de pareja (58,8%) es superior al 48,2% notificado a nivel nacional en 2016; sin embargo, la proporción de mujeres que buscaron ayuda formal (7,1%) es considerablemente inferior al 20% nacional^16^. En esta comunidad, todas las mujeres encuestadas que recurrieron a ayuda formal estaban en dinámicas de terrorismo íntimo, lo cual concuerda con estudios previos^30^, y contrasta con los testimonios de las autoridades locales tanto civiles como religiosas, quienes refieren que las mujeres que han buscado ayuda con ellos no viven situaciones de violencia graves. Esta búsqueda de ayuda en las comunidades resulta en multas o acuerdos conciliatorios sin un seguimiento adecuado, lo que puede poner a las mujeres en riesgo de sufrir más violencia como represalia, incluido el feminicidio^31^. 

Se ha reportado que, en algunos países del Sur Global, es más probable que las mujeres busquen ayuda -tanto formal como informal- cuando el agresor ejerce la violencia en estado de ebriedad^42^. Nuestros resultados muestran que en esta comunidad las autoridades locales solo intervienen si el agresor ocasiona un disturbio público y desestiman la responsabilidad del agresor cuando ejerce violencia bajo estado de ebriedad. Dado que solo un tercio de los casos de violencia de pareja en esta comunidad suceden exclusivamente cuando el agresor está ebrio^4^, centrar los esfuerzos en reducir el consumo de alcohol como medida de prevención de la violencia, resulta insuficiente. El hecho de que solo el 3,8% de las mujeres buscaran ayuda ante autoridades locales, todas sobrevivientes de terrorismo íntimo, podría reflejar la falta de confianza en su capacidad de proporcionar asistencia sin incrementar el riesgo de represalias o vulnerarlas económicamente mediante el pago de multas. Además, las autoridades locales resguardan el orden patriarcal^54^ y mantienen la lealtad entre los hombres al perdonar la multa y promover procesos de conciliación cuando ellos lo solicitan, lo cual dificulta la respuesta adecuada a la violencia de pareja. Adicionalmente, al formar parte de la comunidad y, en algunos casos, ser incluso familiares, recurrir a las autoridades locales podría exponer a las víctimas ante terceros y aumentar la vergüenza, principales razones por las que muchas deciden no compartir sus experiencias de violencia.

Coincidente con lo reportado a nivel nacional (79%), la mayoría de las mujeres buscaron ayuda informal, principalmente de familiares; en contraste, solo el 7,1% reveló la violencia a amistades, frente al 44,1% reportado en la ENDIREH 2016^16^. Lo anterior coincide con que más de la mitad de las mujeres reportó no tener a alguien en quien confiar sus problemas. Esto podría explicarse por normas de género que limitan las interacciones sociales, además de ser una comunidad pequeña, donde muchas personas son familiares. Aunque la solidez de las redes de apoyo no parece estar relacionada con la búsqueda de ayuda en casos de violencia de pareja^30^, esto puede diferir en comunidades rurales donde las redes de apoyo informales son la principal fuente de ayuda.

Este estudio muestra cómo las múltiples formas de violencia que experimentan las mujeres se entrelazan para obstaculizar la búsqueda de ayuda. Por un lado, la violencia estructural, responsable de la pobreza, impunidad y dificultad para acceder a las instituciones de justicia y, por otro, la violencia comunitaria y familiar que no solo las aísla y las culpa, sino que también las responsabiliza de poner fin a la violencia^53^. Estos diferentes tipos de violencias que se ejercen sobre los cuerpos de las mujeres ejemplifican la naturaleza simbólica de la violencia, como lo sostiene Rita Segato^54^, que busca mantener el orden de género patriarcal. En este estudio se demuestra cómo las distintas formas de apoyo que reciben las mujeres de sus redes sociales en parte retan y a la vez permiten que este orden social se sostenga al mitigar el daño, pero difícilmente ponerle fin. El estado, con las leyes patriarcales que desde la década de 1930 otorgaron la tierra a los “hombres de familia” y sus herederos varones, se introduce^54^, en esta comunidad, a través de la estructura política de la Asamblea Ejidal^55^, donde las mujeres, a pesar de resultar electas o poder heredar tierra hoy en día, difícilmente participan.

Por otro lado, las mujeres no son pasivas frente a la violencia, sino que constantemente resisten o responden a esta y reconocen su daño. De hecho, reflexionando sobre la violencia que sufrieron en la infancia y la que actualmente viven, muchas mujeres se posicionan en contra de la violencia, incluso cuando se ejerce como represalia por incumplir roles o normas de género. Nos oponemos a la idea de que las mujeres no buscan ayuda porque normalizan la violencia. Las sobrevivientes de violencia situacional relatan mantener diálogos reflexivos y negociaciones con sus parejas y familiares buscando romper la herencia transgeneracional de la violencia y construir una vida pacífica para ellas y sus hijos e hijas. Las sobrevivientes de terrorismo íntimo evitan estos diálogos al considerarlos provocativos, debido al miedo a la violencia severa por la que han sentido en riesgo de morir. No obstante, trabajan de forma activa en su supervivencia y seguridad a través del trabajo, el resguardo, las alianzas con sus hijos y la separación. Los esfuerzos en México dirigidos a concientizar a las mujeres sobre la violencia de género y sus efectos en la salud, buscando que ellas le pongan un fin^53^, pueden hacerlas sentir avergonzadas y culpables de no poder detenerla. Asimismo, los esfuerzos por “empoderar” a las mujeres rurales para que denuncien, sin mecanismos que garanticen su protección ni la transformación de las masculinidades tradicionales, no solo podrían aumentar el riesgo de violencia en represalia, especialmente para aquellas que sufren terrorismo íntimo^56^, sino que invisibiliza el origen estructural de la violencia de género y cómo esta se entrelaza con otros sistemas de opresión como el capitalismo y el colonialismo^54^.

En nuestras entrevistas queda claro cómo este “nuevo” conocimiento, producto de los esfuerzos de concientización en relación con la violencia hacia las mujeres, permea en la comunidad, replicando la idea de que denunciar es la solución a la violencia, aunque solo el 3,8% acudió a instancias gubernamentales. Además de barreras socioeconómicas y geográficas que dificultan la denuncia, para muchas mujeres separarse de su pareja implica perder la única fuente de ingresos y limitar su capital social. Dado que la mayoría busca ayuda dentro de sus familias y personas cercanas, es importante que estas personas tengan no solo información, sino también habilidades y herramientas prácticas que les permitan identificar cuándo se trata de terrorismo íntimo para extender ayuda de forma adecuada, ya sea dentro o fuera de sus comunidades^42^.

Por otro lado, la agencia de las mujeres no consiste solo en resistirse a las normas de género, sino también en decidir cómo habitarlas^45^,^57^. Las entrevistas muestran que las mujeres desean tener una familia unida y libre de violencia, estar presentes para sus hijos e hijas aun cuando esto implique dedicarse al hogar, que las autoridades cumplan los acuerdos cuando denuncian la violencia, tener personas en las que puedan confiar y sentirse valoradas y aceptadas dentro de su familia y comunidad. Estos deseos demuestran la importancia de contar con redes de apoyo sólidas, con opciones de justicia restaurativa a nivel comunitario y mejores mecanismos de apoyo institucional, que faciliten el acceso y garanticen la seguridad de las sobrevivientes. 

El estudio de la búsqueda de ayuda ofrece perspectivas clave sobre cómo las intervenciones de salud comunitaria pueden abordar la violencia de género y sus consecuencias para la salud, especialmente porque muchas mujeres buscan un espacio confidencial para compartir sus experiencias. Aunque en este estudio solo una mujer mencionó haber expresamente buscado ayuda de un proveedor de salud, el vivir violencia de pareja se expresa en el espacio clínico en el contexto de la atención a la salud mental^49^^,^^50^; es posible que más participantes hayan comentado con alguien la situación de violencia de pareja sin considerarlo búsqueda de ayuda. Además, no se preguntó específicamente si las mujeres buscaban ayuda con las trabajadoras de salud comunitaria que colaboraban en ese momento con Compañeros en Salud. Estas trabajadoras son mujeres locales que reciben supervisión y capacitación continua para atender y prevenir algunos problemas de salud, incluyendo salud mental. A diferencia de las personas profesionales de la salud, entienden el contexto local, pueden dar una atención más culturalmente sensible e identificar casos de violencia dentro de sus comunidades, al igual que recursos locales para manejarla.

Estudios previos en esta localidad han mostrado una prevalencia de depresión del 10,1% entre las mujeres en las clínicas de atención primaria^48^, y un aumento del 6,6% en el riesgo de síntomas depresivos graves entre las mujeres receptoras de terrorismo íntimo^4^. Por lo tanto, el tamizaje de la violencia de pareja entre las mujeres con síntomas depresivos, por parte de profesionales de la salud y de las trabajadoras de salud comunitaria, podría abrir un espacio seguro para que las mujeres hablen sobre su experiencia, tal y como se ha demostrado en otros países^58^^,^^59^. Esto es especialmente importante en el caso del terrorismo íntimo que se asocia con depresión e intentos de suicidio y no recibir asistencia puede incrementar este riesgo^4^.

Tanto las personas profesionales de salud como trabajadoras comunitarias pueden recibir formación en escucha activa, trabajo informado en trauma, planes de seguridad para la violencia y recursos institucionales, con una perspectiva social crítica. Tener un enfoque que no juzgue y reconozca la complejidad de la violencia de pareja es necesario para generar confianza en las sobrevivientes^60^. Del mismo modo, las trabajadoras de salud comunitaria pueden facilitar la búsqueda de ayuda visitando a las mujeres en sus hogares y en otros espacios comunitarios sin que se tengan que desplazar a una clínica de salud. Las intervenciones psicosociales grupales, como los círculos de mujeres, diseñados y dirigidos por trabajadoras comunitarias de salud en las zonas rurales pueden ser una forma culturalmente segura de fortalecer las redes de apoyo y el bienestar emocional, al tiempo que se cuestionan las normas de género^61^^,^^62^. No obstante, las normas de género en comunidades pequeñas podrían dificultar que algunas mujeres se sientan cómodas al comentar la violencia con personas de su comunidad, por lo que es necesario contar también con proveedores de salud externos para ampliar sus opciones de búsqueda de ayuda. Estos espacios podrían ser especialmente útiles para sobrevivientes de terrorismo íntimo, quienes describen mayor aislamiento y, debido a los comportamientos controladores inherentes a esta dinámica de violencia, tendrían mayores dificultades para participar en actividades grupales^62^.

Además, es importante contar con intervenciones de salud mental dirigidas a infancias y adolescencias, ya que sus esfuerzos por mitigar los riesgos de la violencia de pareja pueden repercutir en su desarrollo físico y emocional^63^^,^^64^. Estas intervenciones deben incluir medidas que garanticen la seguridad y la salud mental de las personas proveedoras, especialmente de las trabajadoras de salud comunitaria, ofreciéndoles supervisión, formalización laboral y los recursos necesarios. También es crucial reconocer que, debido a la naturaleza del trauma y sus implicaciones sociales, no todas las mujeres desean revelar sus experiencias, por lo que podrían ofrecerse cuidados de salud mental que no requieran hablar sobre ellas. Al mismo tiempo, profesionales de salud deben reflexionar críticamente sobre el riesgo de medicalizar la violencia, lo que la puede individualizar y despolitizar^64^^,^^65^. Estos esfuerzos deben ir acompañados de cambios sistémicos en las estructuras políticas, sociales y económicas que perpetúan la violencia.

### Limitaciones

Para priorizar un análisis profundo, los datos se recogieron en una pequeña comunidad de Chiapas en una zona predominantemente no indígena. Por lo anterior, estos resultados no son generalizables al México rural, aunque muchas comunidades rurales en México comparten la organización ejidal y características socioeconómicas. Además, la redacción de algunas preguntas de la encuesta cuantitativa puede haber influido en las respuestas, aun cuando el cuestionario fue revisado por personas de la región. Finalmente, la primera autora había trabajado antes como médica en la comunidad, lo que puede haber creado un sesgo de deseabilidad social.

## Conclusión

Este artículo ilustra que en esta comunidad rural la mayoría de las mujeres receptoras de violencia de pareja hablan de ella y buscan ayuda. Sin embargo, buscaron ayuda formal en menor proporción que a nivel nacional, asociado con barreras socioeconómicas, impunidad y minimización de la violencia por parte de las autoridades, así como la falta de acceso a instituciones resolutivas. La mayoría busca ayuda dentro de sus redes sociales, sobre todo con familiares, quienes proporcionan apoyo de diferentes maneras según el género y el parentesco. Por lo tanto, esfuerzos basados en la comunidad que fortalezcan las redes de apoyo y busquen reducir y poner fin a la violencia son necesarios, al mismo tiempo que se mejora el acceso a las instituciones de justicia especializadas. Finalmente, futuras investigaciones sobre la violencia de pareja deben dar prioridad a estrategias metodológicas que garanticen una representación adecuada de los entornos rurales.
