# Supplementary material for: Intimate partner violence and help-seeking in Chiapas, Mexico: Implications for rural community health services
Source: Salud Colect. 2025 Nov 4;21:e5623. [Article in Spanish] doi: 10.18294/sc.2025.5623 (PMC13198630; doi:10.18294/sc.2025.5623)
Supplement: Translation

# Translation: Intimate partner violence and help-seeking in Chiapas, Mexico: Implications for rural community health services

**Authors:** Mercedes Aguerrebere, Ana Cecilia Ortega, Rocío López, Sonia M. Frías

**Affiliations:** 1 Corresponding author. Physician, Specialist in Psychiatry. Consultant in Psychiatry, Compañeros en Salud México A.C., Partners in Health Mexico. Chiapas, Mexico. Researcher, Department of Global Health and Social Medicine, Harvard Medical School. Boston, United States of America. maguerrebere@pih.org; 2 Master’s Degree in International Development. Coordinator, Mental Health Program, Compañeros en Salud México A.C., Partners in Health Mexico, Chiapas, Mexico. aortega@pih.org; 3 Medical Surgeon. Escuela de Medicina y Ciencias de la Salud. TecSalud, Monterrey, Mexico. rociolopzb@gmail.com; 4 Doctor of Sociology, Regional Center for Multidisciplinary Research, Universidad Nacional Autónoma de México. Morelos, Mexico. sfrias@crim.unam.mx

**Keywords:** Rural Health, Intimate Partner Violence, Gender Perspective, Poverty, Mexico

## Abstract

From a gender perspective, this study examines how women seek help in response to experiences of intimate partner violence, distinguishing between dynamics of intimate terrorism and situational violence in a rural community in Chiapas, Mexico. A probabilistic study was conducted between July and August 2017 with the participation of 141 women aged 15 years and older, using an adaptation of the National Survey on the Dynamics of Household Relationships (ENDIREH). In addition, from July to December 2017, in-depth interviews were conducted with intentionally selected women and local leaders, along with participant observation. Among survivors, 59% disclosed the violence, and only 7.1% sought formal help, mainly those who experienced intimate terrorism. Most sought help from their families, and 78.9% felt supported by them; this support included emotional assistance, confronting aggressors, and protecting women. Emotions such as fear and shame, impunity, traditional gender norms, socioeconomic factors, and restricted social networks inhibit help-seeking. In this context, community health programs can play a key role in strengthening social networks and providing care.

## Introduction

Violence against women is globally recognized as a serious public health issue and one of the most systematic and widespread violations of human rights. Intimate partner violence (IPV) consists of harmful actions of a physical, sexual, psychological, or financial nature within any kind of romantic relationship, including manipulative behaviors.^1^ Beyond causing physical harm, IPV is associated with a higher risk of depression and anxiety, post-traumatic stress disorder, and suicide attempts.^2^^,^^3^^,^^4^^,^^5^^,^^6^^,^^7^^,^^8^^,^^9^ IPV must be understood in relation to the multiple forms of violence experienced by different groups of women,^10^ from an intersectional perspective that considers the diverse systems of oppression that shape their lives, including racism, classism, and ableism.^11^ It should also be analyzed in connection with structural violence, understood as the harm caused by global and local political and economic forces that lead to unequal access to resources, services, rights, and safety.^12^^,^^13^ Thus, although IPV cuts across ethnic, cultural, and socioeconomic categories, it affects women differently depending on their context and their available resources to confront it.^11^

According to the 2021 National Survey on the Dynamics of Household Relationships (ENDIREH), 39.9% of women in Mexico have experienced IPV at some point in their lives, and 20.7% in the past 12 months.^14^ The state of Chiapas, where 51% of the population lives in rural areas,^15^ reports the lowest prevalence of IPV in the year prior to the survey (12.6%), with higher rates in urban areas than in rural ones.^14^ This trend is consistent with data from previous editions of ENDIREH.^16^ However, Chiapas ranks lowest nationwide on the Gender Inequality Index, based on calculations using the United Nations Development Programme (UNDP) methodology,^17^ which is theoretically linked to IPV.^18^^,^^19^ Gender inequality in this state is further shaped by other systems of stratification: 67.4% of the population lives in poverty, 28.2% in extreme poverty,^20^ and 28.2% of the population speaks an Indigenous language.^15^

Although the low reported levels of IPV in rural areas of Chiapas may be partly due to methodological issues related to the cultural relevance, administration, and language of survey instruments,^21^^,^^22^ the case of the community analyzed in this article stands out. In this highly marginalized, non-Indigenous rural community of approximately 1,200 inhabitants, located in the Frailesca region of Chiapas, a mixed-methods study conducted in 2017 revealed alarmingly high levels of physical and sexual violence compared to national and state figures.^4^ In the 2016 ENDIREH, carried out one year prior to this study, 16.1% of women in Chiapas reported having experienced physical or sexual violence by their current or most recent partner.^16^ In contrast, in this non-Indigenous rural community, two out of three women (66.4%) reported such experiences.^4^

Given the multiple consequences of IPV on physical and mental health,^2^^,^^3^^,^^4^^,^^5^^,^^6^^,^^7^^,^^8^^,^^9^^,^^23^^,^^24^ it is crucial to examine the dynamics and patterns of help-seeking among women affected by IPV in marginalized contexts. In Mexico, health services could play a key role not only in the prevention and care of IPV but also in referring women to justice institutions, social work, family support, and other relevant services, as established by the Official Mexican Standard on Family, Sexual, and Gender Violence, known as NOM-046.^25^

### Help-seeking strategies and dynamics of intimate partner violence

Intimate partner violence (IPV) is not a homogeneous phenomenon. Some dynamics stem from a desire for domination and control, while others arise from the accumulation of tension that eventually leads to violence. It is therefore important to distinguish between two forms of IPV: *intimate terrorism* and *situational violence*.^28^^,^^29^ The first refers to a general pattern of controlling behaviors rooted in male power and is associated with severe physical and mental health consequences for women, including femicide.^30^^,^^31^ At the population level, 1.2% of all Mexican women over the age of 15 experience this form of intimate terrorism.^30^ The second, situational violence, results from tension and conflict not linked to patriarchal control; nationally, 5.8% of women fall into this category.^30^

In the population studied in this article, both dynamics have been previously documented, revealing considerably higher figures for both types of violence compared to national data, with a greater prevalence of intimate terrorism (40.6%) than situational violence (25.8%).^4^

The health consequences are also more severe among those who experience intimate terrorism. In this same community, intimate terrorism is associated with a 6.6 times higher risk of severe depressive symptoms and twice the risk of suicidal ideation.^4^ Moreover, help-seeking patterns vary depending on the type of violence experienced. Data from ENDIREH 2016 show that the percentage of women who sought formal help for IPV - from public institutions, including health services - is nearly three times higher among those in situations of intimate terrorism than among those experiencing situational couple violence (16.8% vs. 5.5%).^30^

Women are not passive victims: they develop coping strategies by recognizing their vulnerability and mobilizing symbolic, material, and relational resources.^32^ Disclosing their experiences of violence to someone can be understood as a way of seeking help, since it involves an active effort to obtain support to end the violence, as well as recognition, understanding, and emotional accompaniment.^32^^,^^33^ The help-seeking process can be either formal (through public or community institutions) or informal (through members of their support network).

The decision to disclose violence is influenced by the recognition of the violent act itself and by the possibility and willingness to seek help.^34^ Both individual and structural factors shape this decision. For instance, based on ENDIREH 2021 data,^14^ 4.6% of women over 15 who have been physically assaulted by their partners consider such incidents to be of little importance; this percentage rises to 7.2% among those who have been tied up by their partners. Additionally, this process involves assessing potential risks in relation to one’s material, relational, economic, social, and institutional resources.^35^ In Mexico, for example, health care responses after episodes of violence are often inadequate.^36^^,^^37^ Thus, when women perceive help-seeking as unimportant after experiencing violence, this may reflect not only that the violent event itself was considered minor, but also that seeking help was deemed unnecessary, ineffective, or not a priority.

Although traditional approaches to intimate partner violence (IPV) focus on severe physical and sexual violence and on encouraging women to report it to justice, public security, or victim assistance institutions, only 8.3% of survivors seek help from public institutions.^30^ This proportion may be even lower in rural communities due to geographic and economic barriers, as well as local understandings and community-based ways of addressing violence.

Even when women have access to government institutions, many do not seek formal help because they fear retaliation from their partners, family members, or community members; fear not being believed; feelings of shame and guilt; victim-blaming attitudes from society and service providers; and general distrust of institutions.^30^^,^^38^^,^^39^^,^^40^^,^^41^ Consequently, women often turn to informal sources of help. A study across 31 Global South countries found that 34.9% of women experiencing IPV primarily sought help from family members. Help seeking was higher among women experiencing severe IPV, women who had witnessed IPV in their childhood and women with greater economic resources.^42^ In small communities, social belonging also facilitates informal help-seeking.^43^ Although informal help does not always end the violence, it can improve access to information and strengthen social and economic support.^35^^,^^44^

However, many women are reluctant to disclose violence within their social networks due to lack of family support or because they perceive violence as inevitable or justified by gender roles.^40^^,^^45^^,^^46^ Nationally, 41% of women who report IPV to public authorities do not disclose it to their families, mainly due to cultural mandates rooted in *familismo* that reproduce patriarchal structures.^46^ This is particularly relevant in small communities, where social support networks are crucial in emergencies, providing access to money or transportation, and may be fractured if women disclose violence perpetrated by members of those same networks.^47^

Undoubtedly, the case of this rural community can be considered an extreme case.^26^ While it cannot be generalized,^27^ it offers valuable insights into help-seeking behaviors, the types of help required, and the factors that inhibit or prevent women from seeking formal assistance. This study illustrates the social, cultural, and structural factors that shape help-seeking, focusing on how different dynamics of intimate partner violence influence women’s decisions to seek help. Building on this understanding, we analyze the potential role of community health interventions in facilitating both formal and informal help-seeking, as well as in providing care and resources to improve survivors’ health. This is particularly relevant given the limited literature on help-seeking in rural areas of Mexico, where this research can contribute to strengthening rural health services from a gender perspective.^25^

## Methods

This article draws on a convergent mixed-methods study conducted between July and December 2017 in a rural community of approximately 1,200 inhabitants located in the Frailesca region of Chiapas.^47^ The community is about five hours by car from the nearest city. Coffee cultivation constitutes the primary occupation for most men in the community. The majority of residents are Catholic, with smaller groups identifying as Presbyterian or Pentecostal. Community decisions are made during ejidal assembly (*asamblea ejidal*) meetings-the local governing body composed of *ejidatarios*, holders of land rights within the *ejido*, a legally recognized form of collective land tenure with individual or family use in Mexico.^36^ At the time of the study, only 5 of the 85 *ejidatarios* were women. During these assemblies, local authorities (the president, treasurer, and secretary of the *comisariado ejidal*, the rural agent, the rural judge, and the oversight council) are elected annually to serve as community leaders. Together, these authorities oversee the administration of the *ejido*, ensure compliance with local agreements and public order, resolve agrarian disputes, represent the *ejido* politically, and report irregularities to municipal authorities.^36^

The non-governmental organization *Compañeros en Salud* (CES) has been working in this community since 2011 in collaboration with the Ministry of Health, providing medical care in ten rural clinics, one hospital, and two maternity homes. This study emerged after health personnel identified depressive symptoms as one of the most frequent reasons for medical consultations among women,^48^ with intimate partner violence identified as one of the main triggering factors.^49^^,^^50^

A simple random probability sample survey was conducted between July and August 2017. A sample size of 159 women aged 15 and older was calculated, using finite population correction. To select participants, households were first enumerated and randomly chosen from a list provided by the local health center. Within each selected household, all eligible women were ennumerated, and one woman per household was randomly selected. Inclusion criteria were: 1) being a woman, 2) being at least 15 years old, and 3) currently residing in the community. Exclusion criteria were: 1) having a hearing, cognitive, or speech disability, and 2) having another household member participating in the study’s interviews or surveys, to ensure confidentiality. The response rate was 89%. Data collection was carried out by three of the authors and one research assistant.

Questions related to IPV and help-seeking were adapted from ENDIREH. IPV dynamics were classified as *intimate terrorism* when participants reported a high level of partner control (more than four controlling behaviors), physical and/or sexual violence of any severity, or moderate control (between one and four controlling behaviors) combined with severe physical and/or sexual violence. *Situational violence* was defined as the presence of physical and/or sexual violence without controlling behaviors, or with moderate control and low-severity physical and/or sexual violence.^4^

Help-seeking was categorized as *positive* when participants reported having spoken to someone about the violence they experienced, or having sought help from local civil, religious, or health authorities, lawyers, or government institutions. Participants were asked to identify whom they sought help from and what response they received. Quantitative results presented in this article were analyzed using Stata 15, applying descriptive analysis and reporting percentages for categorical variables.

Additionally, to deepen and contextualize the quantitative findings, two of the authors conducted in-depth interviews with ten women (Table 1) and nine key informants. The women were selected based on the variability of their experiences of intimate partner violence, as identified in the survey. The key informants, purposively selected according to their community roles, included five religious authorities (two male and one female Catholic ministers, one Pentecostal pastor, and one Presbyterian pastor) and four local civil authorities (three men - the president of the *comisariado ejidal*, the rural agent, and the representative of the oversight council - and one woman, the municipal syndic).

**Table 1:** Characteristics of women who participated in the in-depth interviews. Frailesca region of Chiapas, Mexico, 2017

Pseudonym	Age	Years of schooling	Intimate partner violence
Eréndira*	34	1	Situational. Witness to intimate terrorism in childhood.
Marisol	36	12	Situational
Yusmi	30	2	Situational. Witness to intimate terrorism in childhood.
Dulce	20	11	None
Juana	26	9	Situational
Rosa	39	9	Intimate terrorism
Romelia	25	9	None
Elba	27	4	Situational
Flori	31	5	Intimate terrorism with previous partner; situational violence with current partner
Guadalupe	37	4	Situational

*The names listed in the table, and throughout the article, are pseudonyms. Only data from women participants are shown, as local and religious authorities could be easily identified due to their role and age.

During data analysis, it was found that both the female catholic minister and the female municipal syndic reported not having received any requests for support related to intimate partner violence. Therefore, the testimonies presented in this article correspond exclusively to the seven male participants.

In-depth interviews and participant observation were conducted between July and December 2017. The interviews explored gender norms and roles, experiences of gender-based violence, help-seeking behaviors, and responses to violence from women themselves, their families, close contacts, and the broader community. Interviews were held in private spaces and lasted between 45 minutes and two hours. All interviews were recorded, transcribed, and analyzed in Spanish. The first author conducted participant observation within the community in a range of social groups and settings (e.g., family gatherings, religious groups, sports tournaments, graduations, Alcoholics Anonymous meetings).

An inductive thematic analysis with a gender perspective was carried out. The first two authors identified codes in the interview sections related to disclosing experiences of violence, seeking help, and the responses received. Thematic categories were then developed through an iterative process of reflection, revisiting the transcripts for clarification and contextual understanding. Any disagreements about codes or categories were resolved by consensus. Ethnographic fieldnotes from participant observation focused on gender norms, beliefs, and expectations related to gender-based violence and help-seeking, and were used to contextualize the qualitative findings.

During the iterative process of qualitative analysis, four themes emerged that informed the contextualization of the quantitative results: 1) limitation of harm, 2) hopelessness in the face of impunity, 3) conflicting gender norms and expectations, and 4) resistance to violence. Ethnographic fieldnotes from participant observation were taken to contextualize the findings. Areas of convergence and divergence between quantitative and qualitative data were identified. Quantitative and qualitative findings are presented in an integrated narrative structured around the four themes derived from the qualitative analysis. Quantitative results demonstrate the scope of the experiences, while qualitative findings illustrate their dynamic context, highlighting opportunities for social and health interventions. Real names were replaced with pseudonyms to protect participants’ identities.

This study followed the World Health Organization’s ethical and safety recommendations for research on violence against women.^51^ Ethical approval was granted by the Office of Human Research Administration at Harvard Medical School (IRB17-0583) and the Chiapas Ministry of Health. Informed consent was obtained from adult participants and assent from adolescent participants for both the survey and the interviews.

## Results

A descriptive analysis was conducted using the responses of the 141 women who completed the survey. Of these, 76.6% were married or in a consensual union, 5% were separated or divorced, and fewer than 10% were single. Nearly one third had entered a union at age 16 or younger, and 90.8% had children. A total of 46.1% had not completed primary school, fewer than half had their own financial resources, and only 22% had access to cash in case of an emergency. Only half of the women reported having someone they trusted to talk to about their problems. Among the 141 women, 66.4% reported having experienced intimate partner violence from their current or most recent partner, 40.6% experienced intimate terrorism, and 25.8% situational couple violence.

The survey results show that four out of ten women in this community experienced violence in silence. Table 2 indicates that 58.8% of the women who reported intimate partner violence disclosed their experience to someone. Only 7.1% (all survivors of intimate terrorism) sought help from institutions or community leaders. Most women sought informal help, in both cases from their family of origin; more survivors of intimate terrorism sought help from their in-laws (21.2%) compared to those experiencing situational couple violence (9.1%). Notably, women in situations of situational couple violence did not seek formal help. It is important to highlight that sharing their experiences with someone did not always result in positive outcomes for the women.

**Table 2:** Formal and informal help-seeking (in percentages), by type of violence dynamic, among women who reported experiencing intimate partner violence. Frailesca region of Chiapas, Mexico, 2017

Variables	Any violence n=85		Intimate terrorism n= 52		Situational couple violence n=33	
	n	%	n	%	n	%
**Disclosed to someone**						
No	35	41.2	21	40.4	14	57.6
Yes	50	58.8	31	59.6	19	57.6
**Formal help (institutional or community)**						
No	79	92.9	46	88.5	0	0.0
Yes	6	7.1	6	11.5	0	0.0
Type of formal help (institutional or community)*						
Government institutions^a^	2	2.4	2	3.8	0	0.0
Local authorities^b^	2	2.4	2	3.8	0	0.0
Religious authorities^c^	0	0.0	0	0.0	0	0.0
Health personnel^d^	1	1.2	1	1.9	0	0.0
Other (lawyer)	1	1.2	1	1.9	0	0.0
**Informal help**						
No	36	42.4	21	40.4	15	45.5
Yes	49	64.6	31	59.6	18	54.5
Type of informal help*						
Woman’s own family	38	44.7	24	46.2	14	42.5
Partner’s family	14	16.5	11	21.2	3	9.1
Friends	6	7.1	4	7.7	2	6.1

Source: Own elaboration. *Multiple responses. ^a^Public Prosecutor’s Office, municipal court, conciliation court, municipal presidency, and the System for Integral Family Development (DIF). ^b^Ejidal commissariat president, rural agent, rural judge, rural secretary, and ejidatarios serving as community police. ^c^Local ministers or pastors. ^d^Physicians or nurses from the community clinic or from outside the community.

### Harm limitation

Most of the women in the survey who sought informal help reported feeling supported, 78.9% of those who sought help from their family of origin, 71.4% of those who sought help from their in-laws, and 66.7% of those who sought help from friends. The support they described was oriented toward limiting harm and can be summarized in four categories identified through the interviews: 1) emotional support, 2) safeguarding the woman, 3) intervening with the aggressor, and 4) children’s intervention. These dynamics of support are shaped by gender roles among those involved: generally, men provide support from a position of authority toward either the aggressor or the woman, while women offer care to the survivors and to children. Lack of support involves not recognizing or minimizing the violence, as well as responding with aggression or blame when the woman discloses her situation.

### Emotional support

Participants described emotional support as one of the most meaningful forms of help, noting relief in being listened to or advised - most often by other women in their family network - regardless of the content of the advice or their willingness or ability to act on it. Survivors of situational couple violence tended to describe more reflective conversations with people close to them, whereas survivors of intimate terrorism more often recounted comments from others (relatives or not) who witnessed the violence.

*My sister would just listen to me, she’d cry with me. Sometimes she would say, “leave him, separate from him, I don’t know, maybe you’ll have a better life,” things like that. And others (my brother-in-law) would say, “don’t leave him”* [...] *In the end, I was the one who had to make the decision* [...] *And well, yes, my family’s support has really helped me move forward*. (Marisol, 36 years old, situational couple violence)

“*Why are you putting up with that old man? Didn’t you find someone younger? Leave him, go take care of your family*.” [...] *My neighbors would sometimes see him hit me, and that’s the kind of advice they gave me, but I would say: and what about my kids?* (Flori, 31 years old, intimate terrorism)

### Safeguarding the woman

Women commonly provided support by protecting the survivor, offering a safe place to stay when her husband “ran out” (*correrse*) of the home, that is, when she fled in fear, usually with her children, to avoid physical violence. Women typically “run out” when they know their husbands have been drinking alcohol and usually become violent while intoxicated. These accounts often came from women talking about their own experiences of intimate terrorism, or from women describing the violence they had witnessed against their mothers. As Yusmi, a 30-year-old woman who experiences situational couple violence and witnessed intimate terrorism in her childhood, explained: “If she [my mother] didn’t run, then my father would have killed her.” Other women described “running out” even when they had not yet experienced physical violence themselves, based on transgenerational knowledge that men become violent when drinking.

*…sometimes we would go stay at one of my aunt’s houses, and since she, her husband doesn’t drink, we’d get there without a blanket or anything, and my mom would just borrow a sack and lay it down on the floor for us to sleep on.* (Eréndira, 34 years old, situational violence, witnessed intimate terrorism against her mother in childhood)

### Intervening with the aggressor

When seeking informal help, different people often place themselves between the aggressor and the woman, most frequently male relatives, since women may be at risk if they intervene. In one interview, for example, a participant noted that when her mother-in-law stepped in between her and her husband, the husband beat her as well. Men tend to threaten the aggressor, reprimand him, or physically restrain him. Physical restraint was described in the interviews only by survivors and witnesses of intimate terrorism. According to the survey data (Table 2), survivors of intimate terrorism sought help from the aggressor’s relatives (21.2%) more often than survivors of situational couple violence (9.1%). This type of support aims to reduce harm during episodes of physical violence and to lessen or stop future violence, although the latter rarely occurs.

*I didn’t do anything to him, like, for him to react that way, right?* […] *No, he just got mad on his own, who knows why* […] *I don’t know if you’ve heard the word engazarse, like when someone gets really worked up, like their nerves just go up and up, and then he’d start throwing whatever he found around, and they’d have to tie him up by his hands and feet, and only then he’d finally fall asleep.* (Rosa, 39 years old, intimate terrorism)

*…they would tell him, I mean, they’d say things to him, but he wouldn’t listen anymore.* […] *Well, his dad would tell him not to act like that, that being married is to get along, not to be fighting and hitting her.* (Rosa, 39 years old, intimate terrorism)

[my dad] *told me, “look, daughter, I’m not going to go to his house because I didn’t hand you over there. Go tell him to come here, I’ll settle things* […] *look, if you don’t love my daughter anymore, fine, maybe that love is over, but talk to her honestly, and don’t keep hurting her.*” (Juana, 26 years old, situational violence)

### Children’s intervention

Children frequently provide instrumental support, particularly in the context of intimate terrorism. Their roles are not strongly gendered, and they provide all three types of support described above. Common strategies include asking their father to stop, distracting him, or helping their mother leave the house. They may also physically restrain the aggressor (putting themselves at risk), seek help, and offer emotional support to their mother. Several interview participants stated that their children’s intervention had saved their lives. It is important to note that all interviewees who recalled witnessing their father’s violence against their mother described these memories with rejection of the violence and with fear.

*And once my sister did defend her, but my dad gave her a hit in the belly and threw she onto the grinder, and that’s how he stayed, and she didn’t come back. And meanwhile we went to call my uncle, and my uncle arrived, and my dad went out to beat my uncle. That was the moment my mom managed to escape, to run away* [correrse] *with all of us.* (Eréndida, 34 years, situational violence; witness of intimate terrorism against her mother in childhood)

*So I told the little girl to help me, and it was the girl who told him, “daddy, let go of my mommy, because you might end up killing her” or something like that. “Only for you will I do it, I’ll let your mom go because I already hate her and don’t want to see her here in the house, but only because you’re asking me, I’ll let her go.”* (Rosa, 39 years, intimate terrorism)

### Lack of support

The women interviewed described, often with pain, situations in which they not only did not receive support from their relatives but were also subjected to emotional mistreatment. According to the survey, 21.1% of the women who disclosed the violence to members of their own family and 28.6% of those who sought help from their in-laws did not feel supported. Among them, some reported mistreatment, referring specifically to being ignored (10.3% by their own family; none reported this from their in-laws), or to being humiliated, not believed, or blamed for the violence they experienced (2.6% by their own family; 14.2% by their in-laws).

In addition, disclosing the violence to family members was hindered by fear of their parents’ reactions, either because of previous experiences of physical or verbal abuse from parents, which was common among the women interviewed, or due to anticipated disapproval of the relationship.

*Once he had already left me really hurt here, and even my mother-in-law came. And she didn’t come to defend me, she came to throw more fuel on the fire, she came to tear me down and defend him* [my husband] […] *Then I went to my mom’s place and my mom yelled at me like always, and I’d say to myself, “this isn’t a life for me, because I get out of the coals just to fall into the flames.” There was no peace anywhere.* (Eréndida, 34 years, situational violence; witness of intimate terrorism in childhood)

*There was one time I left my husband and I started telling my mom* […] *and she says to me, “maybe what you’re saying isn’t even true”* […] *“oh please, when have I ever seen you all beaten up.”* (Juana, 26 years, situational violence)

*I got pregnant again about a year after my first child. He started drinking and I would go to my dad, all sad, and he’d say to me, “look, I told you, but you wouldn’t listen, I told you, look.*” (Elba, 27 years, situational violence)

### Hopelessness in the face of impunity

The low rate of institutional help-seeking shown in Table 2 can be explained in part by socioeconomic and geographic barriers that limit women’s ability to access such services. The community is located one to three hours by car from the nearest public prosecutor’s office. There is no cell phone signal, and internet access is limited, expensive, and available only in public spaces. Half of the women surveyed are economically dependent on their husbands and/or in-laws, and fewer than half have someone who could care for their children if they needed to leave the community. In addition, formal complaints rarely result in compliance with agreements or in the cessation of violence. Among the women surveyed, two sought support from governmental justice institutions; in one case, the partner was fined, and in the other, a conciliatory agreement was reached. The following testimony illustrates the outcome of such an agreement:

*In that time I filed a complaint against him* [my husband]. […] *It went all the way to the local peace court* […] *I filed it because he had hit me and all that. We came to an agreement that he would pay child support and all that, which he never did. And yeah… he did hit me.* (Marisol, 36 years, situational violence)

On the other hand, seeking help from local authorities does not guarantee women’s safety either. The consequences that are typically imposed include a fine paid to the ejidal assembly (which may be waived and can further impoverish families), conciliatory agreements between the couple, temporary community detention (usually less than 24 hours), or the issuance of a statement of facts. Therefore, it is not surprising that so few women turn to them (Table 2).

*Well, here, it depends, sometimes in the community, it depends on the assembly* […] *They set a fine of, like, 500 pesos. Right? For, for the ones who drink, you know.* […] *A lot of people think the fine is harsh.* […] *And sometimes even us, like as authorities, we don’t always enforce the fine the way we should.* […] *Sometimes, since we’re human, I think, you know, friendship between men shouldn’t be broken.* (Religious authority who previously served as a civil authority)

Flori, 31, who experienced intimate terrorism with a previous partner, described what happened after reaching an “agreement” in the community regarding how long her ex-partner was allowed to take their children away from the community:

*He took them for three days, and now it’s been almost two years* […] *We went to the authorities here, but back then, when he was still around and the kids were still here, they told him that he could take them only with my permission and for a maximum of 15 days, no more than that. But he didn’t care*. (Flori, 31 years, intimate terrorism)

Moreover, local authorities have historically been men. The only female local authority interviewed, the community trustee, reported that she obtained the position unwillingly, after her husband was selected by the ejidal assembly, because “the rule changed, that it had to be a woman.” She also stated that she does not participate in the assembly because “that’s separate, that’s more for men,” illustrating that she was unaware that her position granted her both voice and vote within it. According to the testimonies of the men who served as local authorities, they typically only intervene when the violence becomes a public disturbance or when it is associated with alcohol consumption.

“*And then in June he made that whole scene, and they* [the local authorities] *took him to jail* [...]. *He knew he couldn’t come into the house when he was drunk, no, because I knew how he reacted and I didn’t like it* [...]. *And that day he showed up wanting to come in and I told him no, that he couldn’t, and he started making a scene saying I had a lover and who knows what, kicking the door, trying to force it* [...] *and he opened it, he left the door all busted up.*” (Marisol, 36 years, situational violence)

Interviewer: *What happens when a woman is beaten by her husband, even if he’s not drunk?*

Local authority: “*No, in that case, yes, that’s called mistreatment, like, directly toward the person, and we refer that to the public prosecutor, because they’re the ones who will have to apply the law*.”

Likewise, authorities often respond by downplaying or justifying the violence, reinforcing the notion that intimate partner violence should be handled in the private sphere, and thus discouraging help-seeking. Notably, the authorities interviewed stated that they had not had to deal with “serious” cases of intimate partner violence, whereas the survey data show that only women experiencing intimate terrorism sought help from local authorities (Table 2).

...*there has to be some kind of justification for why the person scolded or got mad at his wife or whatever, but he has to own it when he’s the one who messed up. There are people who do accept it, they apologize, but that’s within the family, you know, not something for the authorities. I haven’t seen any other serious problem around here, just problems or mistreatment or… verbal stuff, like when he suddenly comes home drunk, bangs on the door, runs her out* [la corre]*, but that’s verbal, he doesn’t hit them*. (Local authority)

As discussed earlier, “running one’s wife out” or a woman “running herself out” indicates that she fears severe physical violence. This minimization and condoning of violence, together with the desire to preserve the family unit (reinforced by Christian values), encourages local authorities (both civil and religious) to focus their efforts on facilitating processes of forgiveness and reconciliation in order to avoid the couple’s separation:

*A couple, they were already almost, about to, about to split up, and the husband kept throwing the woman and children out of the house, and they would get scared. We went over there, and they agreed to talk… And well, in the end, they forgave each other.* (Religious authority)

*There are couples who fight, husbands hit their wives when they’re drunk, they get reported, and the women come to the office saying that… well… they don’t want to stay with their husbands anymore because they behave badly and all that. A report is filed, and if the couple - well, the wife - decides she no longer wants to live with him, that’s written down. But then the man goes “no, give me another chance,” so they make an agreement, but they want it in writing that if he does it again, the second time, then the woman will go back to her own home* [they separate]. (Local authority)

### Gender norms and conflicting expectations

Regarding the reasons for not seeking help captured in the survey, Table 3 shows that the main ones were: 1) not wanting others to find out (28.6%), a proportion that was higher among women experiencing intimate terrorism than those experiencing situational violence (33.3% vs. 21.4%, respectively); 2) shame (22.9%); and 3) fear (20%). Moreover, 23.8% of women who experienced intimate terrorism reported that their families persuaded them not to disclose the violence to others or to authorities, in contrast to none of the women who experienced situational violence. Four women (28.6%) who experienced situational violence did not seek help “because it was not important,” compared to only one woman who experienced intimate terrorism. Another significant difference is that among women surviving intimate terrorism, 23.8% did not disclose the violence because they feared being mistreated, blamed, or not believed.

**Table 3:** Reasons for not seeking help among survey respondents who reported experiencing intimate partner violence, by violence dynamics. Frailesca Region of Chiapas, Mexico, 2017.

Reasons	Any intimate partner violence (n=35)		Intimate terrorism (n=21)		Situational violence (n=14)	
	n	%	n	%	n	%
So that others wouldn’t find out	10	28.6	7	33.3	3	21.4
Shame	8	22.9	5	23.8	3	21.4
Fear	7	20.0	3	14.3	4	28.6
Her family convinced her not to	5	14.3	5	23.8	0	0.0
It wasn’t important	5	14.3	1	4.8	4	28.6
Other (her mother treated her badly/blamed her/hit her, they didn’t believe her)	5	14.3	5	23.8	0	0.0
It wouldn’t have made any difference / they wouldn’t believe her	3	8.6	2	9.5	1	7.1
Threats	1	8.9	1	4.8	0	0.0
Because of the children	1	2.9	1	4.8	0	0.0
That person had the right to do it or she deserved it	1	2.9	1	4.8	0	0.0
Because people say women should endure it	1	2.9	1	4.8	0	0.0
She didn’t know she could seek help	0	0.0	0	0.0	0	0.0
She needed money and didn’t have any	0	0.0	0	0.0	0	0.0

Source: Own elaboration.

Interviews clearly showed that the decision to seek help is shaped by gender norms. Local authorities stated that women provoke violence when they fail to fulfill gender expectations, such as not performing domestic tasks, not “attending to” their husbands, and not behaving in a calm and compliant manner. The women interviewed also reported hearing this discourse among their relatives, mostly men. The following testimony from a local authority illustrates when he believes women are “to blame” for being beaten:

*…like when you go work out in the fields and you come back and there’s no food. All that, well, someone’s bound to get upset, and the man comes home hungry and there’s nothing. Sometimes that’s how the problems start…* (Local authority)

Initially, in the surveys, several women denied having experienced intimate partner violence; however, they acknowledged it when asked whether their partner had been violent while under the influence of alcohol. Drinking alcohol is part of hegemonic masculinity and is perceived as something intrinsic to being a man.^52^ In the community, violence associated with alcohol consumption is more tolerated because it is considered a consequence of drinking rather than an intentional act that would be more readily recognized as violence.^47^ This lack of recognition when violence occurs in the context of alcohol intoxication is also reflected in the discourse of local authorities, who believe that men are unable to control themselves while intoxicated. In fact, as a way to prevent violent altercations, the ejidal assembly prohibited the sale of alcohol within the community.

*...the drunk, for example, he came, got carried away and had too many drinks, didn’t know what he said, so* [the violence] *is forgiven because he’s drunk more than anything. But someone who is sober, you have to check with a doctor whether he’s mentally ill because he’s saying things he shouldn’t say*. (Local authority)

At the same time, these traditional gender norms have weakened over time, and it is repeatedly stated that women today “should not endure violence.” As a consequence, experiencing violence can become a source of shame. The conflict between recognizing the violence, the expectation that one “should not put up with it,” and the inability to stop it - combined with religious teachings and gender prescriptions-produces ambivalence among some women.

*I do have a doubt about that. Because, well, psychologists and all those people say that if a woman doesn’t want to* [have sex], *her husband shouldn’t force her. But I go to church, I’m Catholic, and the priest tells us that it’s a woman’s duty. So I’m like, which one is right and which one is wrong?* (Juana, 26 years old, situational violence)

Likewise, there is a scarcity of paid work for women in the community, limited to small businesses that are insufficient to cover basic expenses. Gender roles also restrict women’s participation in agricultural work, further constrained by their caregiving and domestic responsibilities. This economic situation limits women’s possibilities to seek formal help, file a complaint, or separate from their partners.

*A woman can’t go out to work* […] *Well, our husbands don’t let us. And also there’s nowhere for us to work anyway. We just stay home, doing the house chores. The husband is the one who works and brings whatever we’re going to need*. (Flori, 31, situational violence in current relationship, intimate terrorism in previous relationship)

However, the following interviewee - the only one with formal employment - describes that when finances are not an obstacle to separation, the desire to maintain a united family becomes more significant. It is worth noting that many of the women interviewed who grew up without a father expressed longing for fathers to be present in their own children’s lives.

*…I mean, there were so many things, I kept thinking, how… I wasn’t that worried about the money, about how I was going to cover my expenses. What worried me was the moral part, maybe - how my son was going to grow up without affection, without a father’s love, without support…* (Marisol, 36 years old, situational violence)

Lastly, the same gender norms confine women to their homes, limiting their social interaction outside the family. In the community, public spaces are dominated by men, and women going out without a specific purpose is frowned upon. The controlling behaviors characteristic of intimate terrorism further isolate women, who adapt their actions in an effort to avoid physical violence. Many women reported throughout the study that they do not have friends, and only half of the survey respondents stated that they have someone with whom they can share their problems. It is therefore not surprising that only 7.1% of women who experienced any type of violence disclosed it to friends (Table 2).

*When we were dating, he was always watching me. I could never go out, or he wouldn’t let me have friends. And maybe that’s why I don’t like going out now - because, well, I got used to not… to being shut in there, to not going out.* (Rosa, 39 years old, intimate terrorism)

*Well, at least here, since I don’t go out, I don’t really trust people or know who I could trust, so I just… you know, keep it to myself. Sometimes I feel sad, and sometimes I feel happy - I don’t even know why - but I’d like to share it with someone, only I can’t. Sometimes I tell my husband, “hey, look, this happened to me, or I want this, or look at this,” but he dismisses me.* (Flori, 31 years old, intimate terrorism in a previous relationship; situational violence with current partner)

This final testimony highlights women’s desire to share what they feel. After completing the survey, some women mentioned that they wished they could talk to psychologists (none are available in the community) or to women doctors. However, Flori, one of the interviewees, explained that although she considered telling her story to the doctor at the clinic, she could not bring herself to do it: “it’s hard, it hurts to tell our story.” During data collection, many women expressed gratitude for the space to be heard and said they felt relieved to finally speak about things they had not been able to “let out.”

### Resistance to violence

Not seeking help does not mean that women remain inactive or that they fail to recognize the violence. Women who describe experiences of situational couple violence attempt to set boundaries with their partners (and relatives) through reflective dialogue, threats, or physical force. In doing so, they challenge traditional gender mandates (even when internalized) and try to break cycles of transgenerational violence. Their goal is to build, for themselves and their children, a life different from the one they experienced growing up. As Flori says, “a good life, full of calm, peace, and love.” In their narratives, they speak of not wanting to “put up with it” like their mothers did, of believing that they are capable of raising their children on their own (especially those who had previously worked in the city), of having told their partners that separation was a real possibility, and of discussing with them how their own past experiences shape their current behavior.

*…I got it into my head that if he hits me, I’ll hit him back. I mean, I just can’t take it anymore - because of my nerves, I can’t stand the idea that he’s going to hit me and I’ll just stand there with my arms crossed, no.* (Flori, 31 years old, situational violence in current relationship; intimate terrorism in previous relationship)

*Sometimes I think and I say to myself, well, I provoked him, he was drunk, so maybe I provoked him. But then I tell myself no, he has no right to hit me.* (Marisol, 36 years old, situational violence)

[I told my husband] *you need to be doing well yourself at home first, as the head of the household, because one day my children will grow up, they’ll see you hitting their mother, others will see them hitting their wife - what are you going to tell them, with what right…? Because you also drink and you’re violent. Before my children grow up, you have to change… Because it’s embarrassing, people notice*. (Eréndida, 34 years old, situational violence)

On the other hand, the women who survived intimate terrorism reported avoiding provoking the aggressor, even in the face of ongoing humiliation, insults, and threats, because they feared the severe violence from which they had already come close to dying. These women expressed feeling alone and described locking themselves away to cry because, as they said, “there was nothing else I could do.”

## Discussion

This study shows that the percentage of women who seek either formal or informal help in situations of intimate partner violence (58.8%) is higher than the 48.2% reported at the national level in 2016. However, the proportion of women who sought formal help (7.1%) is considerably lower than the national rate of 20%.^16^ In this community, all the women who sought formal help were experiencing intimate terrorism, which is consistent with previous studies.^30^ This contrasts with the testimonies of local authorities - both civil and religious - who claimed that the women who had sought help from them were not experiencing severe violence. Seeking help through community mechanisms and within the ejidal community tends to result in fines or conciliatory agreements without adequate follow-up, which can place women at increased risk of retaliatory violence, including femicide.^31^

It has been reported that in some countries in the Global South, women are more likely to seek help - formal and informal - when the aggressor perpetrates violence while intoxicated.^42^ Our findings show that in this community, local authorities only intervene when the aggressor causes a public disturbance and tend to diminish the aggressor’s responsibility when violence occurs under the influence of alcohol. Given that only one-third of intimate partner violence cases in this community occur exclusively when the aggressor is intoxicated,^4^ focusing prevention efforts primarily on reducing alcohol consumption is insufficient. The fact that only 3.8% of women sought help from local authorities, all of whom were survivors of intimate terrorism, may reflect a lack of trust in the authorities’ ability to provide assistance whithout increasing the risk of retaliation or the risk of causing further economic harm through fines. Additionally, local authorities uphold patriarchal order^54^ and maintain loyalty among men by waiving fines and promoting conciliatory processes when requested by them, which hinders an adequate response to intimate partner violence. Furthermore, because authorities are members of the same community, and in some cases family members, seeking their help may expose women to further social scrutiny and shame, which are among the main reasons many women choose not to disclose violence.

Consistent with national reports (79%), most women sought informal help, primarily from relatives. In contrast, only 7.1% disclosed the violence to friends, compared to 44.1% reported in the 2016 ENDIREH.^16^ More than half of the women also reported that they did not have anyone to confide in. This may be due to gender norms that restrict women’s social interactions, as well as the small size of the community, where many people are relatives. Although the strength of social support networks does not appear to be associated with help-seeking in cases of intimate partner violence overall,^30^ this may be different in rural communities where informal support networks are the primary source of help.

This study shows how the multiple forms of violence that women experience intersect in ways that hinder their ability to seek help. On the one hand, structural violence produces poverty, impunity, and barriers to accessing justice institutions. On the other, community and familial violence not only isolates and blames women, but also holds them responsible for ending the violence.^53^ These overlapping forms of violence enacted on women’s bodies exemplify the symbolic nature of violence described by Rita Segato,^54^ which serves to maintain patriarchal gender order. Our findings demonstrate that the different forms of support women receive from their social networks both challenge and simultaneously sustain this social order: they may mitigate harm, but rarely stop the violence. The state, through patriarchal laws that since the 1930s granted land to the “family man” and his male heirs, is embedded^54^ in this community through the political structure of the Ejido Assembly,^55^ where women, despite now being eligible to inherit land or be elected to local office, still rarely participate.

At the same time, women are not passive in the face of violence. They actively resist, respond to, and recognize its harm. Reflecting on the violence they witnessed in childhood and the violence they currently experience, many women position themselves against it, even when violence is used as a punishment for not complying with gender roles. We counter the idea that women do not seek help because they normalize violence. Survivors of situational couple violence describe engaging in reflective dialogue and negotiation with partners and relatives, seeking to break the transgenerational transmission of violence and build a peaceful life for themselves and their children. Meanwhile, survivors of intimate terrorism avoid such dialogue because they fear that it will provoke severe violence from which they feel their lives are at risk. Even so, they work actively toward survival and safety through income generation, seeking shelter, forming alliances with their children, and separation. Efforts in Mexico aimed at raising awareness about gender-based violence and its effects on health, framed around the idea that women should end the violence themselves,^53^ can lead women to feel ashamed or guilty when they are unable to do so. Similarly, initiatives to “empower” rural women to report violence, without mechanisms that ensure their protection or that address the transformation of traditional masculinities, may increase the risk of retaliatory violence, particularly for those experiencing intimate terrorism,^56^ while also obscuring the structural origins of gender violence and its entanglement with capitalism and colonialism.^54^

Data from the qualitative interviews make it clear that this “new” knowledge - produced through awareness efforts related to violence against women - circulates within the community, reinforcing the idea that reporting is the solution, even though only 3.8% of women sought help from state institutions. Beyond socioeconomic and geographic barriers that hinder reporting, separation often entails losing the household’s primary source of income and reducing women’s social capital. Because most women seek help within their families and close networks, it is crucial that these networks not only have information, but also practical skills to identify when violence constitutes intimate terrorism and to provide appropriate support, either within or outside the community.^42^

Moreover, women’s agency does not only lie in resisting gender norms, but also in deciding how to inhabit them.^45^ The interviews show that women desire a united and nonviolent family, to be present for their children even if this means dedicating themselves to domestic work, that authorities uphold agreements when violence is reported, to have people they can trust, and feel valued and accepted by within their families and community. These desires highlight the importance of strong support networks, community-based restorative justice options, and improved institutional support mechanisms that ensure access while guaranteeing survivors’ safety.

The study of help-seeking offers key insights into how community health interventions can address gender-based violence and its consequences for health, particularly because many women seek a confidential space to share their experiences. Although in this study only one woman explicitly mentioned having sought help from a health provider, intimate partner violence often emerges in clinical settings in the context of mental health care.^49^^,^^50^ It is possible that more participants discussed the violence they were experiencing with someone without identifying that as help-seeking. Furthermore, the study did not specifically ask whether women sought help from the community health workers who were collaborating at the time with *Compañeros en Salud.* These workers are local women who receive ongoing supervision and training to address and prevent certain health issues, including mental health. Unlike formally trained health professionals, they understand the local context, can offer more culturally sensitive care, and are able to identify cases of violence within their communities, as well as local resources to address it.

Previous studies in this region have shown a 10.1% prevalence of depression among women attending primary care clinics,^48^ and a 6.6% increase in the risk of severe depressive symptoms among women experiencing intimate terrorism.^4^ Screening for intimate partner violence among women presenting with depressive symptoms, carried out by both health professionals and community health workers, could open a safe space for women to talk about their experiences, as has been demonstrated in other countries.^58^^,^^59^ This is particularly important in cases of intimate terrorism, which is associated with depression and suicide attempts; lack of support can increase this risk.^4^

Both health professionals and community health workers can receive training in active listening, trauma-informed care, safety planning for violence, and the use of institutional resources, all from a critical social perspective. A non-judgmental approach that acknowledges the complexity of intimate partner violence is necessary to build trust among survivors.^60^ Likewise, community health workers can facilitate help-seeking by visiting women in their homes or other community spaces, reducing the need for them to travel to a clinic. Group-based psychosocial interventions, such as women’s circles, designed and led by community health workers in rural areas, can create a culturally safe means to strengthen support networks and emotional well-being while questioning gender norms.^61^^,^^62^ However, gender norms in small communities could make some women uncomfortable discussing violence with people from their community, which highlights the need for external health providers to diversify help-seeking options. These spaces may be especially useful for survivors of intimate terrorism, who report greater isolation and, due to the controlling behaviors inherent in this form of violence, may find it more difficult to participate in group activities.^62^ It is also important to implement mental health interventions aimed at children and adolescents, since their efforts to mitigate the risks of intimate partner violence can affect their physical and emotional development.^63^^,^^64^

These interventions must include measures to ensure the safety and mental health of providers, especially community health workers, by offering supervision, labor protections, and adequate resources. It is also crucial to recognize that, due to the nature of trauma and its social implications, not all women wish to disclose their experiences, and mental health care that does not require direct discussion of them should be available. At the same time, health professionals should critically reflect on the risk of medicalizing violence, which can individualize and depoliticize it.^64^^,^^65^ These efforts must be accompanied by systemic changes in the political, social, and economic structures that perpetuate violence.

### Limitations

To prioritize an in-depth analysis, data were collected in a small community in Chiapas located in a predominantly non-Indigenous area. Therefore, these results are not generalizable to rural Mexico as a whole, although many rural communities in the country share *ejido*-based land organization and similar socioeconomic characteristics. In addition, the wording of some survey questions may have influenced responses, even though the questionnaire was reviewed by individuals from the region. Finally, the first author had previously worked as a physician in the community, which may have created a social desirability bias.

## Conclusion

This article shows that in this rural community, most women experiencing intimate partner violence talk about it and seek help. However, they sought formal assistance less often than the national average, which appears to be related to socioeconomic barriers, impunity and the minimization of violence on the part of authorities, as well as limited access to responsive institutions. Most women seek help within their social networks, particularly from family members, who provide support in different ways depending on gender and kinship roles. Therefore, community-based efforts that strengthen support networks and work to reduce and end violence are necessary, alongside improved access to specialized justice institutions. Finally, future research on intimate partner violence should prioritize methodological strategies that ensure adequate representation of rural settings.
